# Targeting the redox system for cardiovascular regeneration in aging

**DOI:** 10.1111/acel.14020

**Published:** 2023-11-13

**Authors:** Meret Sarah Allemann, Pratintip Lee, Jürg H. Beer, Seyed Soheil Saeedi Saravi

**Affiliations:** ^1^ Center for Molecular Cardiology University of Zurich Schlieren Switzerland; ^2^ Department of Internal Medicine Cantonal Hospital Baden Baden Switzerland; ^3^ Center for Translational and Experimental Cardiology, Department of Cardiology University Hospital Zurich, University of Zurich Schlieren Switzerland

**Keywords:** cardiovascular aging, cardiovascular regeneration, cellular senescence, redox machinery, stem/progenitor cell

## Abstract

Cardiovascular aging presents a formidable challenge, as the aging process can lead to reduced cardiac function and heightened susceptibility to cardiovascular diseases. Consequently, there is an escalating, unmet medical need for innovative and effective cardiovascular regeneration strategies aimed at restoring and rejuvenating aging cardiovascular tissues. Altered redox homeostasis and the accumulation of oxidative damage play a pivotal role in detrimental changes to stem cell function and cellular senescence, hampering regenerative capacity in aged cardiovascular system. A mounting body of evidence underscores the significance of targeting redox machinery to restore stem cell self‐renewal and enhance their differentiation potential into youthful cardiovascular lineages. Hence, the redox machinery holds promise as a target for optimizing cardiovascular regenerative therapies. In this context, we delve into the current understanding of redox homeostasis in regulating stem cell function and reprogramming processes that impact the regenerative potential of the cardiovascular system. Furthermore, we offer insights into the recent translational and clinical implications of redox‐targeting compounds aimed at enhancing current regenerative therapies for aging cardiovascular tissues.

## Graphical abstract

Aging is linked to impaired cardiovascular regeneration, with age‐related redox imbalances promoting senescence pathways in cardiac and endothelial stem/progenitor cells. These mechanisms attenuate the intrinsic regenerative potential of these cells and diminish the effectiveness of regenerative interventions in the cardiovascular system. Redox regulators hold the potential to augment the efficacy of cardiovascular rejuvenating strategies in older individuals.

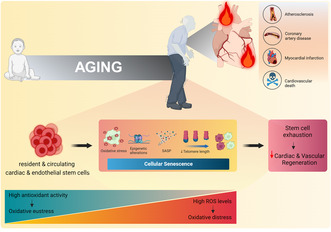

**Abbreviations**
AAD: aging‐associated diseases
AGE: advanced glycation end products
AP: apurinic/apyrimidinic
APE1/Ref‐1: apurinic/apyrimidinic endonuclease1/redox factor‐1
CM: cardiomyocyte
CO: carbon monoxide
CoPP: cobalt protoporphyrin
CP‐312: cardioprotectant‐312
CPC: cardiac progenitor cells
CSC: cardiac stem/progenitor cells
CVD: cardiovascular disease
DHA: docosahexaenoic acid
EC: endothelial cell
ECFC: endothelial colony‐forming cell
eNOS: endothelial nitric oxide synthase
EPA: eicosapentanoic acid
EPC: endothelial progenitor cells
ESC: embryonic stem cells
FoxO: forkhead box
GPx: glutathione peroxidase
Grx: glutaredoxin
GWAS: genome‐wide association study
H_2_O_2_
: hydrogen peroxide
H_2_S: hydrogen sulfide
HGPS: Hutchinson–Gilford progeria syndrome
HIF‐1α: hypoxia inducible factor‐1α
HO‐1: heme oxygenase‐1
I/R: ischemia/reperfusion
iPSC: induced pluripotent stem cells
mECT: mitochondrial electron transport chain
MEF: mouse embryonic fibroblasts
MI: myocardial infarction
mPTP: mitochondrial permeability transition pore
NAC: N‐acetyl‐L‐cysteine
NLRP3: nod‐like receptor protein 3
NO: nitric oxide
NOX: NADPH oxidase
Nrf2: nuclear factor erythroid 2‐related factor 2
Nrp1: neuropilin 1
PM_2.5_
: particulate matter
Prx: peroxiredoxin
PUFA: polyunsaturated fatty acids
ROS: reactive oxygen species
SASP: senescence‐associated secretory phenotype
SDF‐1: stromal cell‐derived factor‐1
SMPC: smooth muscle‐like progenitor cell
SOD: superoxide dismutase
SRF: serum response factor
t‐BHQ: *tert‐*butylhydroquinone
Trx, Txn: thioredoxin
TrxR: thioredoxin reductase
VEGF: vascular endothelial growth factor
VSMC: vascular smooth muscle cells

## 1 CARDIOVASCULAR AGING AND DISRUPTED REGENERATIVE CAPACITY

Lifespan has nearly doubled over the recent seven decades, but the final years of life come often with aging‐associated diseases, most prominently cardiovascular disease (CVD) featured by progressive deterioration of cardiovascular structure and function (Kubben & Misteli, 2017; Paneni et al., 2017). CVD claims about half of all human deaths worldwide and exponentially increases as age advances (Tsao et al., 2022). Aging imposes extensive changes on cardiovascular tissues that lead them toward a pathological state including hypertrophy, left ventricular dysfunction, arterial stiffness, and vascular dysfunction (Donato et al., 2018; Gude et al., 2018; Ungvari et al., 2018). Extrinsic factors, such as environment and lifestyle, and intrinsic processes, such as oxidative stress and inflammation, exacerbate DNA damage response, metabolic remodeling, and epigenetic drift, and thereby promote cellular aging in the cardiovascular system (Gude et al., 2018). These irreversible changes progressively impair the ability of cells to proliferate, which is critical to replace damaged cells that naturally accumulate in aged cardiac and vascular tissues (Guo et al., 2022). During the recent decade, it is increasingly understood that the accumulation of the non‐proliferating cells, so‐called “senescent cells,” declines mammalian tissues and organ function (Owens et al., 2021; Tchkonia & Kirkland, 2018). According to the emerging “adult stem cell senescence theory of aging,” stem cells and/or progenitor cells harboring in the heart and blood vessels or circulating progenitor cells, which replenish either preexisting senescent stem cells or specialized cardiomyocytes (CMs) and endothelial cells (ECs), become exhausted and lose their stemness during aging (Smith & Daniel, 2012). The aging/senescence milieu suppresses endogenous regenerative and reparative mechanisms in the adult stem cells and progenitor cells, and also limits the success of cell‐based regenerative therapies that aimed at repairing injured and dysfunctional tissues and restoring a youthful phenotype in the cardiovascular system (Cianflone, Cappetta, et al., 2020; Cianflone, Torella, et al., 2020). Accordingly, in a middle‐size human study involving 119 humans with cardiovascular disease (32–86 years), more than 50% of tissue‐specific cardiac progenitor cells (CPCs) exhibited senescence phenotype including replicative arrest, telomere attrition, and the senescence‐associated secretory phenotype (SASP) in the infarcted heart of aged patients undergoing cardiac surgery (Lewis‐McDougall et al., 2019). It becomes more important when disruption of cardiovascular repair system develops aging‐associated diseases (AAD) and causes mortality in elderly (North & Sinclair, 2012). Yet, a limited level of evidence exhibits that AAD, namely Type II diabetes mellitus (T2DM), can affect the biology of multipotent CPCs and promotes cellular senescence, per se, by upregulating pathologic SASP, including pro‐inflammatory cytokines (IL‐1α, IL‐1β, and IL‐6) and chemokines (MCP‐1 and MMP‐3), and suppress adult myocardial regeneration (Rota et al., 2006; Shakeri et al., 2018). Therefore, the secret to successful regenerative strategies stands on expanding our knowledge of the nature and mechanisms underpinning cardiovascular aging and regenerative competence of stem/progenitor cells during aging.

**FIGURE 1:**
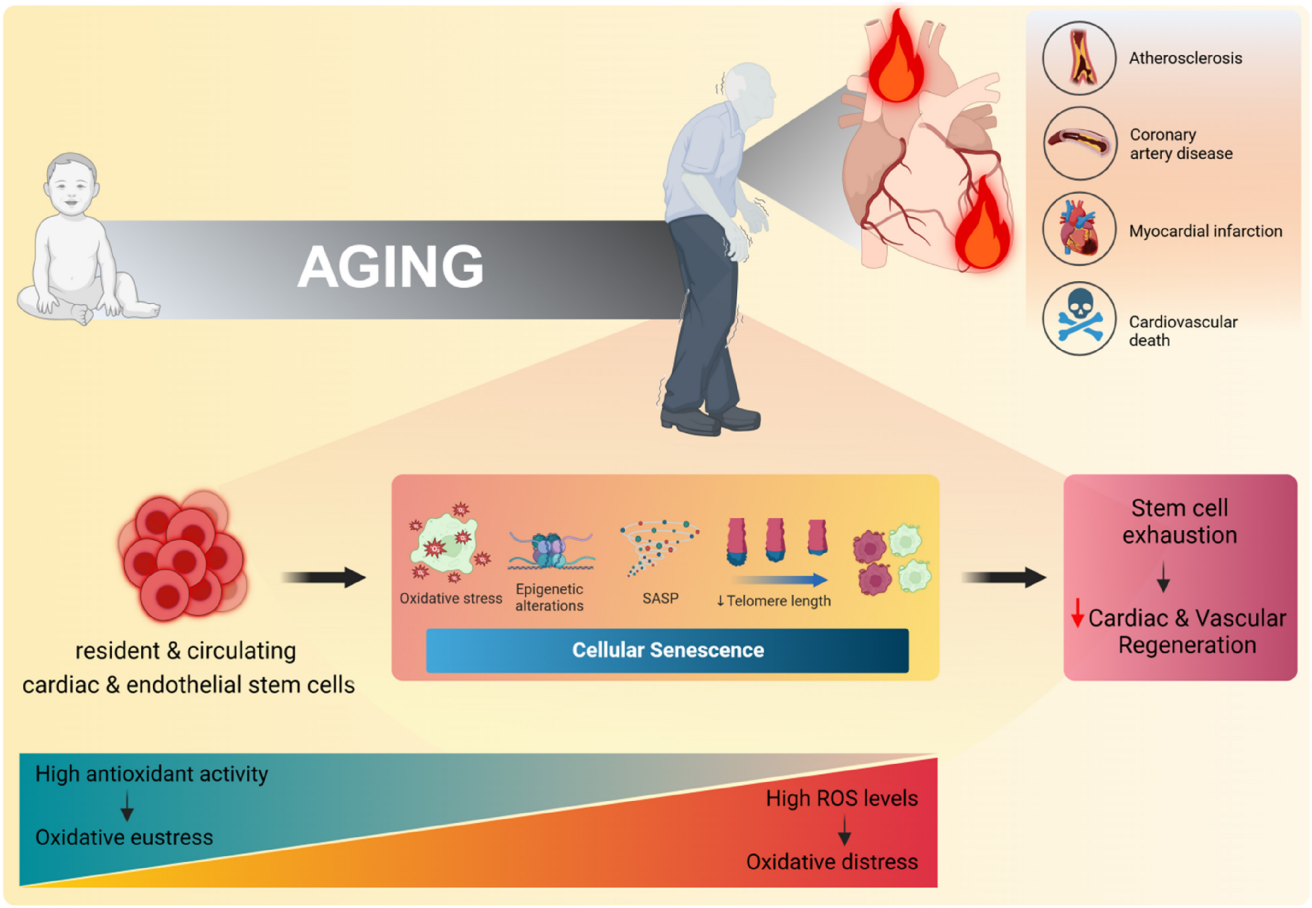
Schematic representation of disrupted cardiovascular regeneration due to age‐related redox imbalance in cardiac and endothelial stem cells. Images created with BioRender.com.

Cardiovascular aging is considered as a consequence of accumulation of cellular senescence‐promoting mechanisms including oxidative stress, mitochondrial injury, genetic and epigenetic modifications, telomere shortening, and metabolic dysregulation (Song et al., 2020; Figure 1). Reactive oxygen species (ROS) have been viewed as pathological molecules that undermine normal cellular pathways by increasing oxidative stress (Eroglu et al., 2019; Saeedi Saravi et al., 2020). The cardiovascular system is principally vulnerable to ROS‐induced oxidative damage due to its high metabolic demand and low antioxidant defense capacity in aging (Sorrentino et al., 2019; Xie et al., 2023). Accordingly, single‐cell RNA‐Seq analysis of mouse aged cardiovascular ECs reveals transcriptomic reprogramming, including upregulation of reactive oxygen species metabolic process in these cells (Gou et al., 2022). In *Macaca fascicularis* monkeys, scRNA‐seq of aortas also shows an inactivation of forkhead box O3A (FOXO3A) in arterial ECs that recapitulates the major phenotypic defects observed in aged monkey aortas. It verifies age‐related loss of FOXO3A as a key regulator of endothelial aging (Zhang, Murugesan, et al., 2020; Zhang, Zhang, et al., 2020). Notably, FOXO3A has been recognized as an evolutionarily conserved transcription factor that acts in redox regulation and correlates with longevity (Klinpudtan et al., 2022) and a lower prevalence of cardiovascular disease in long‐lived humans (Ronnebaum & Patterson, 2010). Not only in aged arterial ECs, single‐nucleus RNA‐Seq verify that oxidative responses are enriched in aged CMs in both primate (Ma et al., 2021) and human (Koenig et al., 2022) hearts. These studies and beyond have demonstrated that these aged cardiac and arterial tissues exhibit a higher level of senescence‐associated β‐galactosidase staining and expression of pro‐senescence genes including IL1β, IL17, and Type‐I interferon (IFN‐α). The relentless ROS production can also cause oxidative stress in cellular components, leading to cardiovascular stem/progenitor cell senescence and impaired proliferation and differentiation (Nishimura et al., 2019; Suzuki & Shults, 2019). These cells exhibit a developed senescence characteristics, including telomere attrition, activation of the *p53*/*CDKN2A*/p16^*INK4A*^/*Rb* molecular pathways, chromatin remodeling, and secretion of a complex mixture of SASP (such as pro‐inflammatory cytokines and chemokines, growth and angiogenic factors, matrix metalloproteinases, nonprotein small molecules, and bioactive lipids) (Cianflone, Cappetta, et al., 2020; Cianflone, Torella, et al., 2020). Indeed, specific transcription factors including GATA4 (Kang et al., 2015), NF‐κB (Ito et al., 2017), mTOR (Laberge et al., 2015), p38MAPK (Freund et al., 2011), and Notch1 (Hoare et al., 2016) signaling molecules, which upregulate during aging, may activate SASP in the stem cells. In both physiological and premature aging, excessive ROS compromise complex proteins regulating the synthesis of bioenergetics in mitochondria, subsequently encompassing disruption of energy homeostasis necessary for cell proliferation and regeneration (Kubben & Misteli, 2017; Picca et al., 2018). In this way, ROS may not only damage adjacent organelles in stem/progenitor cells, but also disrupt widespread metabolic pathways controlling their self‐renewal (Oh et al., 2014). In addition, imbalance of the triad of metabolic signaling‐mitochondrial integrity‐ROS coupling aggravates autophagy, an evolutionarily conserved process which governs clearance of aged/senescent cells containing damaged macromolecules and defective mitochondria (Aman et al., 2021; Ren & Zhang, 2018). These result in a decline in both cardiovascular stem/progenitor cell pool and function and protective pro‐survival autophagic recycling of senescence cells, through which repair and regeneration of damaged cardiac and vascular tissues are overwhelmed (Wiley & Campisi, 2021).

In contrast to excessive ROS, antioxidant defense and hypoxic niche are crucial to maintain stem cells in quiescence state and protect them from cellular senescence (Suzuki & Shults, 2019). What we encounter in aging is downregulation or incompetence of antioxidant defense mechanisms, including ROS‐neutralizing enzymes (Tan et al., 2018). In particular, superoxide dismutase (SOD), mitochondria‐targeted catalase, and glutathione peroxidase (GPx) decrease in these cells with aging (Mandraffino et al., 2012; Spyropoulos et al., 2022). The levels of endogenous antioxidant molecules, such as glutathione and vitamin C, also decline as age advances. Furthermore, aging contributes to both downregulation of stressor‐activated transcription factors such as the nuclear factor erythroid 2‐related factor 1 (Nrf1) and 2 (Nrf2) and its target genes, which regulate the expression and activity of an array of antioxidant enzymes (Dai et al., 2012; Kubben et al., 2016). Gene Ontology (GO) analysis in human‐induced pluripotent stem cell (hiPSC)‐derived CMs revealed that Nrf1 protects these cells against ROS‐induced oxidative damage by the proteasomal activity and antioxidant response, including activation of the detoxifying enzyme heme oxygenase‐1 (HO‐1) (Cui et al., 2021). In line with the reduced antioxidant response observed in aging, there is a significant increase in the expression of inflammatory genes, such as nod‐like receptor protein 3 (NLRP3), which suppresses SOD activity and triggers cellular senescence (Ding et al., 2022). Wang and colleagues also have shown that accumulation of these deleterious mechanisms in endothelial progenitor cells (EPCs) reduce their angiogenic capacity and repair potential in ischemic and damaged blood vessels (Wang et al., 2019). The aged heart and vasculature exhibit the accumulation of oxidative damage and disrupted antioxidant defense mechanisms in cardiovascular stem/progenitor cells, with concomitant tissue remodeling and organ dysfunction (Izzo et al., 2021).

These suggest that restoration of a balance between prooxidants and antioxidants in these cells may potentially regulate their cell cycle and metabolic and epigenetic signals that control self‐renewal and function to preserve regenerative capacity and combat aging‐related CVD (Dudek et al., 2021; Maraldi et al., 2021; Rampon et al., 2018). Accumulated clues to understanding complex redox machinery in aging cardiovascular stem/progenitor cells present a chance to explore novel optimized cardiovascular regenerative strategies which could be life‐saving for elderly patients. With an eye on future, in this review, we focus on the recent findings on the roles of ROS and redox machinery in cardiovascular stem cell senescence in aging, as well as advances in interventions targeting redox signaling for optimal cardiovascular regenerative therapies and clinical potential for the healthspan expansion.

## 2 CARDIOVASCULAR REGENERATION: CURRENT STRATEGIES AND PITFALLS

For decades, cardiovascular tissues, particularly the heart, have been considered as non‐regenerative organs by converging data showing that cardiac and vascular cells are indeed terminally differentiated cells with no replication competence (Cianflone, Cappetta, et al., 2020; Cianflone, Torella, et al., 2020; Salerno et al., 2022). In addition, a wealth of reports swiftly declares that no cardiac stem/progenitor cells (CSCs) exist in the adult tissues to generate new CMs. On this premise, it is clear that spontaneous regenerative capacity on its own is slow and insufficient to repair injured tissues and restore their function in physiological aging and in pathological states (Salerno et al., 2022). The long‐standing paradigm of the adult stem cells and progenitor cells with no endogenous regenerative activity has become more critical by researches remarking the regenerative and reparative decline throughout life (Ellison et al., 2012). It is well established that cardiovascular stem and progenitor cells are subject to age‐associated alterations that progressively impair regenerative mechanisms in the heart and vasculature as age advances (Ballard & Edelberg, 2007). Accordingly, aging memory, oxidative stress, inflammation, and epigenetic modifications, which are known to drive cellular senescence, are attributed as major barriers of regenerative therapies in aging (Hashimoto et al., 2018; Maldonado et al., 2023). These limitations have remained cardiovascular mortality rates unchanged in old populations, despite of considerable progress in prognosis and treatment of cardiovascular disease over the past decades (Roth et al., 2020). Clearly, there is an urgent need for a compelling strategy for cardiovascular repair and regeneration.

**FIGURE 2:**
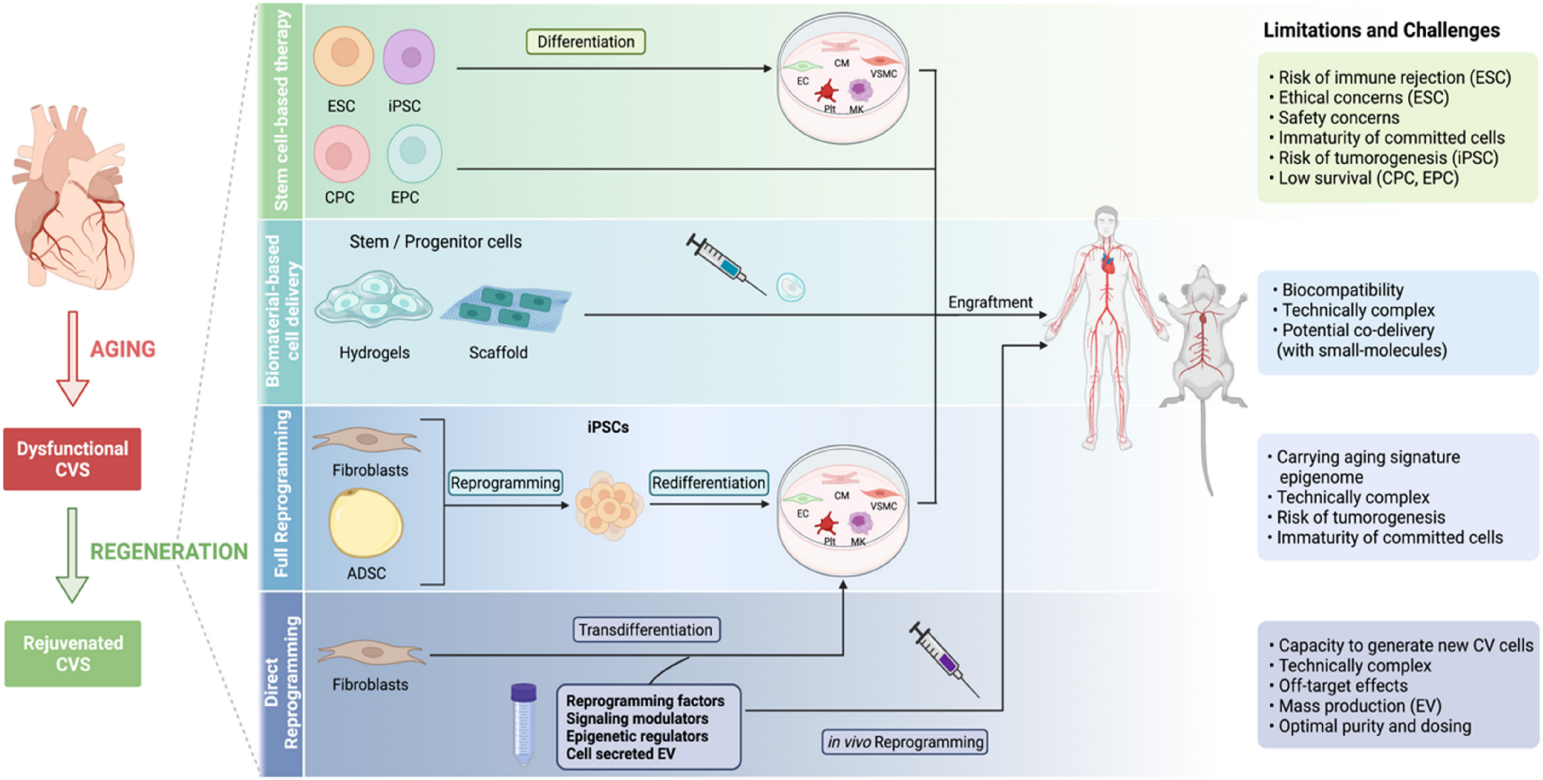
The potential and challenges of cell‐based cardiovascular regenerative therapies. Cell‐based strategies for cardiovascular regeneration by (i) direct engraftment or (ii) biomaterial‐based delivery of CV cells derived from iPSC, ESC, or cardiac and endothelial stem/progenitor cells, as well as (iii) full reprogramming and (iv) direct reprogramming of fibroblasts or ADSCs. In vivo cell‐free direct reprogramming of non‐CV cells toward CV cells, particularly cardiac myocytes, opens a new avenue to overcome the existing limitations and challenges of full reprogramming and other cell‐based therapies. These strategies are or can potentially be tested by clinical trials for replacement of dysfunctional aged CV cells and the subsequent rejuvenation of the CVS. Images created with BioRender.com.

Since 20 years ago, efforts have been on the basis of cell‐based cardiovascular regenerative approaches at most (Figure 2); however, the outcomes of multiple experimental and clinical trials have been neutral (Menasché, 2018). For instance, administration of stem cells or progenitor cells into either failing heart or injured vasculature exhibits a poor cell survival, because the preexisting oxidative microenvironment causes the cell death by 90% and impairs their function (Sthijns et al., 2018; Tenreiro et al., 2021). Although a few numbers of innovative strategies, namely by using the complex biomaterial‐based cell delivery system, appear to circumvent this limitation and enhance stem cell viability, the current interventions are still insufficient (Mu et al., 2022; Vasu et al., 2021).

By using cellular, genetic, cell transplantation, and molecular means, we and other scientists are attempting to find an efficient strategy to protect cardiovascular cell homeostasis, repair, and regeneration (Ellison et al., 2013). The emergence of hiPSCs stands as a game‐changing breakthrough, sparking widespread enthusiasm for their potential clinical applications (Protze et al., 2019). hiPSCs have been set as an unlimited source for generating functional cardiovascular cells with the potential of autologous transplantation into aging heart and vasculature (Csöbönyeiová et al., 2015; Tenreiro et al., 2021). This discovery constructed a basis for cell reprogramming strategies to overcome many of the immunological and ethical limitations of multipotent embryonic stem cells (ESCs) or mesenchymal stem cells for stem cell‐based therapies (Oikonomopoulos et al., 2018; Salerno et al., 2022). These advantages render iPSC technology an exciting prospect for personalized cardiovascular regenerative therapies (Atchison et al., 2020; Shi et al., 2017). The current two intriguing cell reprogramming strategies include the four pluripotency transcription factors (so‐called “Yamanaka factors”) for iPSC reprogramming or “direct reprogramming” that allow CMs and vascular cells reentry into the cell cycle and restore their youthful‐like function. Of note, it permits a direct derivation of CMs, ECs, or vascular smooth muscle cells (VSMCs) in vivo without any need for an intermediate stage of in vitro reprogramming into iPSCs (Nishimura et al., 2019; Yamakawa & Ieda, 2021). Nevertheless, possible tumor formation and aging‐ or pathologic‐related epigenetic memory limited clinical implications of these approaches (Ma et al., 2017). To this end, hiPSCs have been engineered to control reactivation of the cell cycle and to remove aging memory by epigenetic manipulation (Scesa et al., 2021). Accordingly, a series of complementary cell‐free approaches including spatiotemporal administration and manipulation of the pluripotency transcription factors and specific signaling molecules, microRNAs, epigenetic regulators, and extracellular vesicles, alone or in combination, can potentially be used to optimize reprogramming efficiency (Salerno et al., 2022; Tenreiro et al., 2021).

Despite of the outstanding breakthroughs, there are several key questions to be answered, including the mechanisms underlying the effects of cell therapy for cardiovascular aging and the paracrine mechanisms of neighboring cells, as well as the reasons for poor proliferative ability, low efficiency, and immature function of reprogrammed cells. Basic research may provide the needed answers and help to find true regenerative agents with adjusted dose and time and to optimize combined cell‐based and cell‐free strategies as the best next future therapy for cardiovascular regeneration during aging.

## 3 REDOX SYSTEM: ORCHESTRA CONDUCTOR OF INTRINSIC CARDIOVASCULAR REGENERATION

**FIGURE 3:**
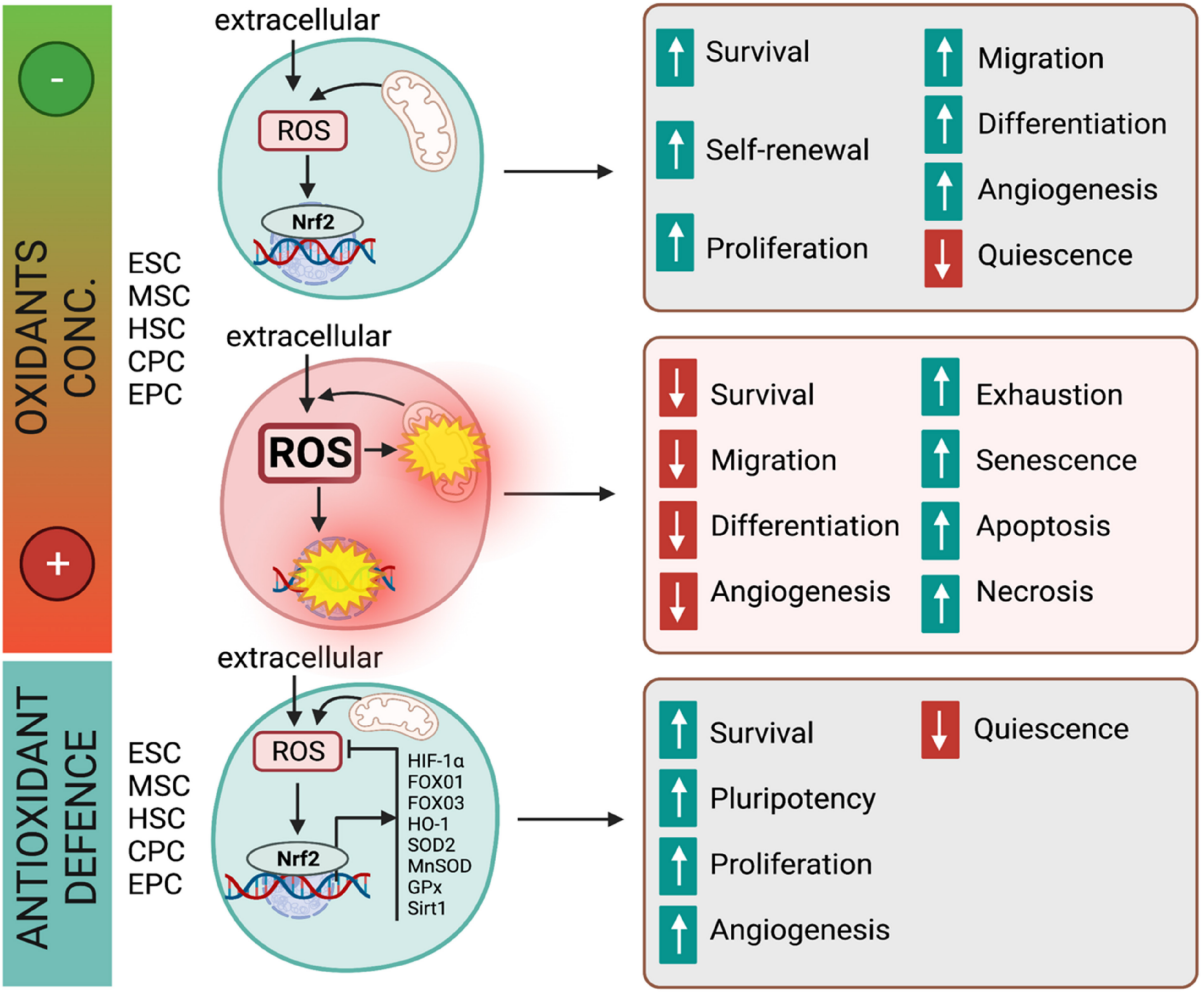
Redox regulation of cardiovascular stem/progenitor cells. Intrinsic redox homeostasis differentially regulates cellular signaling, metabolism, and function in pluripotent/multipotent stem cells (ESC) with potential to differentiate into cardiac and endothelial stem/progenitor cells (CPC and EPC), which guarantee the potential of renewal/repair during aging. (Upper) ROS, at lower levels, are crucial for *stem cell self‐renewal*, proliferation, differentiation, and function, as well as quiescence balance (positive effects). (middle) By contrast, excessive ROS, considered a hallmark of aging and aging‐associated disease, are impair stem cell pool and promote stem cell exhaustion, senescence and apoptosis, which thereby disrupt differentiation to cardiovascular lineages and their function (negative effects). (lower) The antioxidant defense senses intracellular ROS and well‐orchestrates metabolic and epigenetic state and function of the stem cells, although it downregulates with age. Transcription factors Nrf2 and FoxOs, in response to oxidative stress and hypoxia, upregulate cytoprotective genes (e.g., HIF‐1α, HO‐1), scavenging enzymes (e.g., Gpx and SOD), and the histone deacetylases sirtuins, rescue ROS‐induced stem cell senescence and apoptotic death. The antioxidant defense also maintains cell survival, self‐renewal, pluripotency, proliferation, and cardiovascular lineage differentiation. Redox homeostasis facilitates direct differentiation of stem cells into target cardiovascular cells, fostering cardiovascular repair. Images created with BioRender.com.

For decades, ROS were recognized as deleterious molecules encompassing a diverse set of free radicals derived from molecular oxygen that undermine normal cellular pathways (Cui et al., 2012; Kattoor et al., 2017). The “free radical theory of aging” proposes that aging is caused by accumulation of cellular damage inflicted by higher concentrations of ROS, thereby triggering DNA damage and accelerating stem cell senescence (Harman, 2009). Nevertheless, this theory does not tell the whole story (Gladyshev, 2014). Of note, there are surprising controversies that ROS, at lower concentrations, are essential for vital cellular processes contributing to stem cell maintenance and differentiation, particularly in reprogramming strategies (Rampon et al., 2018; Suzuki & Shults, 2019). Yet, as age advances, an accumulation of excessive ROS becomes evident within cells and its persistent presence inflicts damage on cellular components (Tan et al., 2018). Chronic ROS‐induced oxidative stress accumulates genomic instability and declines mitochondrial integrity and cellular proteome that lead to stem cell exhaustion and insufficient regenerative capacity in organisms with progressive CVD (Donato et al., 2018; Gude et al., 2018). For example, genetic deletion of nicotinamide adenine dinucleotide phosphate (NADPH) oxidases 2 and 4 (NOX2 and NOX4), which generate H_2_O_2_, in CPCs can restore their stemness, as inferred by increased expression of c‐kit and vascular endothelial growth factor (VEGF) receptor FIk1 (Maraldi et al., 2021; Momtahan et al., 2019). The concentration‐dependent effects of ROS on stem cells underscore the delicate balance required for redox signaling to exert beneficial or detrimental impacts on cellular aging. Nature has evolutionarily learned to sink ROS signaling by multiple pathways, allowing for the regulation of redox balance throughout life. However, intracellular pathways guaranteeing redox homeostasis in the cardiovascular system and beyond become incompetent with aging (Kimura et al., 2014; Wang et al., 2013). Redox homeostasis is a balance of ROS production and elimination that is crucial for the maintenance of stem cell self‐renewal and function. During the past decade, it is well understood that redox homeostasis guarantees tissue regeneration and reparative reserve to tackle aging‐related loss of function (Figure 3) (Kubben & Misteli, 2017; Oh et al., 2014). A plethora of evidence suggests that redox homeostasis plays a primordial role in cardiovascular stem cell self‐renewal and in the balance of self‐renewal and differentiation (Hashimoto et al., 2018). Redox homeostasis orchestrates intra‐organelle crosstalk, namely between cell mitochondria and nucleus, thereby coordinating metabolic pathways and epigenetic alterations and perpetuating stem cell pool and differentiation throughout life (Wang et al., 2013).

In order to maintain redox homeostasis, stem cells require activating the enzymatic systems responsible for ROS catabolism and disruption of ROS‐mediated oxidative damage (Daiber et al., 2021). Hence, stem cells exploit diverse strategies which are no less complicated than those enzymatic systems responsible for ROS generation. They increase the expression of antioxidant enzymes or redox‐sensitive transcription factors to maintain the proliferative efficiency of resident stem/progenitor cells and their capacity to differentiate into various cardiovascular cell lineages, including cardiac and endothelial progenitor cells (Korski et al., 2019). For instance, stem cells recruit hypoxia niche and associated transcription factors, namely hypoxia inducible factor‐1α (HIF‐1α), to regulate antioxidant enzymes and thereby protect their pool against hallmarks of senescence. To this, the hypoxic environment induces cell quiescence, the state of reversible replicative arrest in which cells do not temporarily divide and yet retain the metabolic capacity to re‐enter the cell cycle (Kimura et al., 2014). In addition, stem cells activate other cellular protective features such as radical scavenging enzymes. SOD2 is a scavenging enzyme that can buffer oxidants in stem cells. In cultured CPCs, SOD2 has been shown to contribute to cell survival in an oxidative state induced by xanthine oxidase, an enzyme that uses xanthine to yield both superoxide (O2^•−^) and H_2_O_2_ (Seshadri et al., 2012). Thioredoxins are another key antioxidant buffering system that localize to distinct subcellular compartments, in particular the cell mitochondria by thioredoxin 2 (Trx2), and cannot only neutralize ROS but also reverse protein oxidation events by reducing disulfide bonds, thereby maintaining the proliferative capacity of stem cells (Jung et al., 2016; Kameritsch et al., 2021). Interestingly, Trx2 creates a complex with peroxiredoxin 1 (Prx1), an antioxidant enzyme which both buffers oxidants and transfers oxidizing equivalents to cysteine‐containing proteins, in mitochondria to contribute to metabolic regulation of heart and vascular development and in zebrafish (Huang et al., 2017) and mouse (Chen et al., 2017; Kiermayer et al., 2015) models. Thioredoxin reductase 2 (TR2), which recycles Trx2 and regulates Trx system, also plays a central role in the control of vascular integrity as seen in endothelial cell‐specific knockout (*Trxrd2* ^*iECKO*^) mice (Kameritsch et al., 2021). In contrast, deletion of genes encoding TR2 and Prx1 are associated with aging phenotype in EPCs, by upregulating apoptotic BCL‐2/Bax‐Caspase‐3 pathway, and seen in age‐associated CVD (Kameritsch et al., 2021; Nonn et al., 2003). Sirtuin 1 (SIRT1), a NAD^+^‐dependent histone deacetylase, is another key upstream regulator of the expression of antioxidant genes including Mn superoxide dismutase (MnSOD), Trx2 and its recycling regulator TR2, and localizes in the regulatory regions of these genes (Jung et al., 2016; Turinetto et al., 2016). It is proven in cultured EPCs and mesenchymal stem cells (MSCs) at late passages which exhibit replicative senescence phenotype, leading to cell exhaustion. These cells display lower cellular NAD^+^ content accompanied by decreased expression of SIRT1 and SIRT3, thereby aggravating oxidative stress and promoting multiple hallmarks of cellular senescence (D'Onofrio et al., 2015; Zhang et al., 2021). It is essential to acknowledge that these examples tell some, but not all aspects of the intricate interactions between the buffering enzymes to combat oxidative damage; for instance, mitochondrial glutaredoxin 2 (Grx2), a class of thiol oxidoreductases that reduce disulfide bridges by a dithiol mechanism similar to that of thioredoxins, interact with SIRT1 for controlling mitochondrial dynamics and function necessary for embryonic heart formation and vascular development in both human and zebrafish models (Berndt et al., 2014; Bräutigam et al., 2013; Staudt & Stainier, 2012). Grx2‐mediated redox signaling pathway activates SIRT1 through reversible *S*‐glutathionylation of this enzyme on its cysteine residue located in a catalytic domain (Zee et al., 2010). Accordingly, cardiac ablation of another member of this family, Grx3, in young cardiac‐specific Grx3 knockout (Grx3‐KO) mice has been associated with left ventricular hypertrophy and heart failure (HF), while wild‐type mice exhibited lower levels of ROS and thereby normal Ca^2+^ handling in cardiomyocytes (Donelson et al., 2019; Ogata et al., 2021).

The enzymatic antioxidant defense system is regulated by master transcription factors, which drive stem cell‐specific gene expression programs mitigating oxidative stress. The nuclear factor erythroid 2‐related factor 2 (Nrf2) and FoxO family are well studied to regulate stem cell homeostasis, self‐renewal, and pluripotency (Dai et al., 2020; Ronnebaum & Patterson, 2010). Not only multipotent stem cells, but unipotent stem cells such as endogenous CPCs, strictly preserve their redox regulators Nrf2, FoxOs, and downstream‐buffering enzymes Trx and apurinic/apyrimidinic (AP) endonuclease1/redox factor‐1 (APE1/Ref‐1), by which prevent the accumulation of mitochondrial ROS (Korski et al., 2019). Nrf2 also retains biological function of either EPCs or endothelial colony‐forming cell (ECFC) and ameliorates ROS‐induced oxidative stress by activating downstream target genes *Hmox1* (encoding HO‐1) and *Txn* (encoding Trx) in old mice (Wang et al., 2018, 2019). Nrf2 activation restores angiogenic capacity and aggravates re‐endothelialization, which is substantially deteriorated with aging (Gremmels et al., 2017). By contrast, the sequestering of the transcription factors underpins stem cell exhaustion and senescence, thereby promoting premature aging seen in both human and animal models of Hutchinson–Gilford progeria syndrome (HGPS) (Kubben et al., 2016; Villa‐Bellosta, 2020). Consistetly, in CSCs, inhibition of APE1/Ref‐1 is accompanied by increased transcription of HIF‐1α and Nrf2, accumulating excessive ROS that subsequently reduce stem cell viability and reinforce them to senesce (Maraldi et al., 2021). Given the strong literature, we anticipate that boosting antioxidant defense system during aging will permit a persistent cellular redox balance in cardiovascular progenitor cells and tissues and will optimize regenerative therapies. Nevertheless, a few preclinical studies notify about the time‐ and concentration‐dependent effects of exogenous antioxidants. For instance, small‐molecule nonselective NOX inhibitors apocynin or diphenyleneiodonium (DPI), which blunt H_2_O_2_ generation, have been shown to inhibit cardiac regeneration in adult zebrafish (Han et al., 2014). A very similar effect was also conferred by forced overexpression of catalase gene, a key enzyme which catalyzes the decomposition of H_2_O_2_ to water and oxygen (Han et al., 2014; McQuaig et al., 2020). In this model, H_2_O_2_ formation (~30 μM) in the epicardium and adjacent compact myocardium at the resection site primes the heart for regeneration. Mechanistically, H_2_O_2_ generated from Duox/Nox2 destabilizes the redox‐sensitive dual‐specificity MAPK phosphatase 6 (Dusp6) and subsequently increases Erk1/2 phosphorylation to confer its pro‐regenerative effects (Missinato et al., 2018; Zhou et al., 2022). Hence, transgenic overexpression of Dusp6 in Tg(*hsp70:dusp6‐His*) zebrafish results in a decrease in Mef2C^+^/BrdU^+^ proliferating myocytes in hearts and further impairs cardiac regeneration (Han et al., 2014). However, what is seen in aged organisms is a chronic and persistent elevation of ROS in diverse cells and tissues that builds an oxidative microenvironment. Therefore, a ROS‐preconditioning might not stimulate regenerative pathways in aged failing hearts and vasculature; instead, it can adversely affect survival and function of circulating and tissue‐specific CPCs and EPCs in different ways, leading to insufficiency and impaired homeostasis and repair potential in the aged cardiovascular tissues.

The abovementioned complex roles of redox machinery in cardiovascular progenitor/stem cell biology and function briefly provide an insight for better understanding of the yin and yang of redox regulation in the context of cardiovascular aging. Although a ROS‐antioxidants balance is essential for progenitor/stem cell differentiation, aging‐associated oxidative stress and dysfunctional antioxidant defense is an inevitable reality that impairs regenerative capacity in the cardiovascular system. In the following, we discuss the dark side of prooxidants and the homeostatic effects of endogenous antioxidants in EPC and CPC upon aging.

## 4 H_2_O_2_ AND OTHER ROS

Nature has devised multiple sources of ROS, allowing for a number of intracellular pathways regulating physiological metabolism and function (Maron & Michel, 2012). However, excessive activity of these sources upon aging leads to pathological outcomes and further aging‐associated diseases (Saeedi Saravi et al., 2020). This view exemplifies the bipashic dose‐dependent effects of ROS, particularly H_2_O_2_, in cells (Sies, 2014). The intracellular sources comprise H_2_O_2_‐generating enzymes targeted to membrane structures (nucleus and plasma membrane). Principal H_2_O_2_‐generating enzymes include the family of NADPH oxidases, which have distinctive subcellular localization profiles and generate H_2_O_2_ in specific cellular locales (Montiel et al., 2020). Other oxidases, for instance, xanthine oxidases and monoamine oxidases, produce H_2_O_2_ as a byproduct of catabolism of specific metabolites (Furuhashi, 2020). The most important organelle for ROS formation after the nucleus is the mitochondrion, in which electron transport chain contributes the largest portion of basal ROS levels (Finkel, 2011; Holmström & Finkel, 2014). Importantly, mitochondria generate more H_2_O_2_ and other ROS (e.g., O_2_^•−^) when cells become senescent, leading to the accumulation of oxidant products such as glycation end products (AGE) (Chaudhuri et al., 2018). Accordingly, mitochondria of elderly people exhibit lowered ATP biosynthesis and bioenergetics imbalance, Ca^2+^ accumulation, and insufficient antioxidative capacity (Amorim et al., 2022). It largely causes chromatin remodeling and a proliferative arrest, but also a consequence.

It is traditionally well characterized that excess H_2_O_2_, both endogenously and exogenously, promotes cellular senescence, which consequently develop CVD in aged organisms (Hu et al., 2022). Aged humans display elevated markers of oxidative stress in their plasma as opposed to young individuals (Wybranowski et al., 2022). During the past two decades, in the context of cardiovascular aging, it is increasingly unraveled that H_2_O_2_ drives cellular senescence in both circulating and tissue‐resident EPCs and CPCs in aging microenvironment and impairs their function, compromising their regenerative capacity (Han & Kim, 2023; Imanishi et al., 2008). It is of great importance that circulating EPCs have been recognized as significant indicators of overall cardiovascular health and actively contribute to cardiovascular homeostasis. Vasa et al. initially introduced competent circulating EPCs (CD34^+^, KDR (VEGFR2)^+^) at higher levels in healthy humans as compared to coronary artery disease (CAD) patients (Vasa et al., 2001). Converging evidence supports the idea that a reduction and dysfunction of EPCs are positively associated with cardiovascular risk factors such as aging and cardiometabolic diseases. Moreover, aging has been clearly recognized to impair the function of circulating EPCs, for instance, by elevating angiotensin‐II, which provokes ROS production and TNF‐α upregulation (Calò et al., 2011; Endtmann et al., 2011). Consistently, aged senescent EPCs have been observed in atherosclerotic plaques and in arteries of patients with CAD (Vemparala et al., 2013) and diabetes (Song et al., 2017; Tepper et al., 2002; Xiang et al., 2022). Furthermore, oxidized low‐density lipoprotein (oxLDL), a primary contributor to atherosclerosis, has been demonstrated to accelerate the senescence of EPCs (Di Santo et al., 2008). This phenomenon significantly contributes to EC dysfunction, ultimately catalyzing the development of atherosclerosis. In this context, oxLDL‐mediated ROS plays a pivotal role in triggering DNA instability and telomere attrition by inhibiting the phosphorylation of phosphatidylinositol 3‐kinase (PI3K)/Akt/hTERT in EPCs (Lai & Liu, 2015; Ming et al., 2016). Therefore, interventions aimed at reversal of EPC senescence and enhancing its mobilization and proliferation may improve cardiovascular outcomes. Accordingly, some natural compounds, specifically adipose‐derived protein Visfatin (Ming et al., 2016) and *Angelica sinensis* polysaccharides (Lai & Liu, 2015), have undergone in vitro testing to reverse oxLDL‐induced EPC senescence. This reversal is achieved by downregulating gp91^phox^, a subunit of NADPH oxidase complex, and reducing overall ROS‐induced oxidative stress in these cells.

In vitro and in vivo studies showed that H_2_O_2_ at concentrations higher than 30–50 μM induces DNA damage and telomere attrition, the cellular alterations which contribute to stress‐induced premature senescence (SIPS) of these progenitor cells (Chen et al., 2013; Monsanto et al., 2020; Saeedi et al., 2022). Accordingly, prolonged exogenous H_2_O_2_ exposure has been most commonly used as an in vitro model for induction of SIPS, which shares features of replicative senescence. These cells exhibit major hallmarks of cellular senescence including irreversible proliferative arrest, increased SA‐β‐galactosidase positivity, and increased expression of cyclin‐dependent kinase (CDK) inhibitors and the SASP. Moreover, senescent EPCs lose their DNA repair capability that accelerates telomere shortening (Wu et al., 2023). Persistent DNA damage and a decline in its cell–cell adhesion and incorporation into the dysfunctional endothelium have been observed to develop atherosclerosis in old *ApoE* ^−/−^ mice. Instead, administration of bone marrow‐derived EPCs from young mice could reverse atherosclerosis in these mice (Edelberg et al., 2002; Zhu et al., 2007). Although little is known about the mechanisms underpinning H_2_O_2_‐induced EPC senescence, the senescence‐promoting mechanisms of H_2_O_2_ in endothelial and vascular smooth muscle cells are comprehensively uncovered. For instance, H_2_O_2_ triggers SIPS by increasing oxidative stress and production of proinflammatory cytokines IL‐1α and IL‐6, proven markers of the SASP. H_2_O_2_ does it by downregulating the heterogeneous nuclear ribonucleoproteins A1 (hnRNP A1)—Octamer‐binding transcriptional factor 4 (Oct4) complex (a regulator of pluripotency in stem cells that confers protective effects against atherosclerosis) (Han, Bedarida, et al., 2018; Han, Kim, et al., 2018) and NADPH‐producing vasculoprotective pathways, rendering HUVEC and hASMC cells to inhibition of mitochondrial fatty acid β‐oxidation and cellular dysfunction (Kalucka et al., 2018).

An association between ROS, cellular senescence, and T2DM, as a life‐threatening AAD has long been proposed. Diabetes is the common pathological event that connects CVD and aging (Shakeri et al., 2018). Accordingly, diabetic cardiomyopathy has been considered as a cardiac disease of aging, and its development and progression has been strongly associated with accelerated ROS‐induced oxidative stress and mitochondrial dysfunction in cardiac cells (Kayama et al., 2015). Combined relatively low endogenous antioxidant capacity and increased ROS levels in the heart has been also observed in heart failure with preserved ejection fraction (HFpEF), which comprises >50% of all HF cases worldwide (Jasinska‐Piadlo & Campbell, 2023; Saeedi Saravi et al., 2023). In diabetic patients, circulating high glucose and altered insulin signaling are associated with increased ROS generation and susceptibility to oxidative responses in the heart, leading to tissue remodeling, ventricular hypertrophy, and impaired systolic and diastolic function (Huo et al., 2023; Nishio et al., 2004). Analysis of heart‐resident CPCs in insulin‐resistant humans and rodents reveal that hyperglycemia‐induced oxidative stress impairs CM formation and growth (Molinaro et al., 2022; Rota et al., 2006). The CM formation‐death imbalance leads to premature CPC senescence and subsequent cardiac aging and HF in diabetic patients (Westermeier et al., 2016). Accordingly, the c‐kit^+^/CD45^−^/CD31^−^ CPCs exhibit an impaired cell growth and lower myogenic differentiation potential. Transcriptional analysis showed that the cells express significantly less than non‐diabetic CPCs the cardiac transcription factors (including *GATA4*, *NKX2.5*, and *MEF2C*) and myocyte contractile genes (including *TNNT2*, *ACTC1*, *MYH6*, and *MYH7*) during myogenic differentiation. The DM‐CPCs also exhibit the major hallmarks of replicative senescence provoked by exaggerated oxidative stress, including increased SA‐β‐gal‐positive cells and histone γ‐H2A.X phosphorylation, higher apoptotic rate, reduced telomerase activity, and elevated SASP. However, senotherapy using a combination of dasatinib, a specific tyrosine kinases‐targeting drug, and quercetin, the natural flavonoid that targets BCL‐2 family members as well as HIF‐1α, could rescue cellular senescence and proliferative capacity. D + Q significantly overrides senescent cell antiapoptotic pathways (SCAPs) selectively in senescent CPCs and reduces DNA damage and p16^INK4a^–positive nuclei CPCs and CMs in T2DM mice. The senolytic approach in vivo could also downregulate SASP expression and upregulate the main cardiac transcription factors, thereby mitigating myocardial pathologic remodeling and cardiac diastolic dysfunction. For the latter, D + Q improved cardiac repair and regenerative capacity in diabetic mice (Marino et al., 2022). Moreover, in a mouse model of diabetes, *p66shc*‐mediated ROS production displays a critical role in CSC senescence and HF, whereas diabetic *p66* ^*shc−/−*^ mice exhibited a higher number of resident CSC pool in their hearts. Of note, *p66* ^*shc*^ is a catalytic subunit of NADPH oxidases that is essential for superoxide formation (Rota et al., 2006). The diabetic *p66* ^*shc−/−*^ mice also showed a markedly higher survival and function of CSCs and a preserved cardiac function compared to diabetic wild‐type mice. It has been suggested that *p66* ^*shc*^ gene knockout downregulates p53 and p16^INK4a^ and thereby rescues telomere attrition in CSCs. Consistently, targeted mutation of *p66* ^*shc*^ gene reduces both ROS generation and susceptibility of CSCs to oxidative stress, and thereby expands mouse lifespan. These data raise the possibility that diabetic cardiomyopathy is in part a stem cell senescence‐based disease. However, much remain to uncover ROS‐regulated signaling pathways which underpin diabetic cardiomyopathy and strategies that can rescue CPC senescence.

Beyond diabetes, specific chemotherapies of maliognancies have been increasingly associated with cardiomypathies and HF and accelerate aging by approximately 17 years of life span (Wood et al., 2016). Human studies of cancer survivors reveal a higher incidence of HF. For instance, the Swiss Childhood Cancer Survivor Study (SCCSS) has reported a higher risk of developing HF among 5‐year acute lymphoblastic leukemia (ALL) survivors who received anthracyclines such as doxorubicin (Hau et al., 2019). In both humans and animals, ALL chemotherapy induces cardiotoxicity and senescence hallmarks. Doxorubicin‐induced cardiomyopathy has been observed to lead to a significant accumulation of ROS and the premature cellular senescence not only in adult cardiomyocytes (CMs) but also in the endogenous cardiac stem cell (CSC) pool (Piegari et al., 2013). This phenomenon was accompanied by acute activation of pro‐apoptotic pathways and DNA damage, indicated by an increased phosphorylation of γ‐H2A.X in CSCs. In another study involving mice with doxorubicin‐induced cardiomyopathy, CPCs displayed an upregulation of miR‐200c, which directly targets the zinc finger E‐box binding homeobox 1 (ZEB1) binding sites. The reduction in ZEB1 binding was concurrent with an increased expression of SASP, specifically CXCR4, reinforcing cell cycle arrest in these cells. Consequently, doxorubicin depletes myocardial regenerative potential and renders the myocardium more susceptible to failure (Beji et al., 2017). Today, doxorubicin is commonly used for both in vitro model of oncogene‐induced cellular senescence and in vivo model of cardiac aging in basic and translational research (Sun et al., 2022). Interestingly, administration of resveratrol, a natural SIRT1 activator with antioxidant activity, to mice with doxorubicin‐induced cardiomyopathy could prevent excessive accumulation of ROS in resident CSCs and restore their reparative capacity in injured hearts (De Angelis et al., 2015). In addition, consistent outcome was observed in mice with doxorubicin‐induced cardiotoxicity in response to another natural antioxidant compound, *Citrus bergamot* polyphenol‐rich fraction (BPF). Similar to the previous study, ROS depletion and regulation of redox balance and autophagy play a central mechanistic role in survival and functional restoration of c‐kit^+^/CD45^−^/CD31^−^ CSCs in the injured myocardium (Carresi et al., 2018).

Together, age‐related oxidative stress induced by ROS, in particular H_2_O_2_, underpins incompetent cardiac and endothelial regenerative and reparative efficiency in both physiological and pathological states. Pharmacological interventions targeting ROS signaling pathways may hold promise for rejuvenating EPC and CPC populations, offering potential therapeutic strategies for combating cardiovascular aging.

## 5 ANTIOXIDANTS

### 5.1 Nrf2

#### 5.1.1 In EPCs

Nrf2 is a ROS‐responsive transcription factor encoded by the *NFE2L2* gene in mammalians. It acts as master regulator of stem cell redox and metabolic homeostasis in stem cells (Kubben & Misteli, 2017). Although Nrf2 regulation in chronological and premature aging and metabolic diseases, namely T2DM, is well studied, the role of Nrf2 signaling cascades in cardiovascular stem cells and tissue regeneration with advanced aging is not still clear. Concerning the fact that adult stem cells miss their function and regenerative capacity throughout life, targeting Nrf2 may be a key approach to provide a balance between suppressing and driving mechanisms for cardiovascular tissue‐specific ASC proliferation, turnover, and self‐renewal. Accumulating data reveal that Nrf2 can protect ASC in the presence of chronic stressors, including oxidative stress, and thereby maintain their limited regenerative capacity during aging (Dai et al., 2020; Wang et al., 2018). Accordingly, in an in vivo model of hindlimb ischemia, studies show that Nrf2 and its downstream antioxidant target genes *Ho‐1*, *Nqo‐1*, and *Trx* are downregulated in EPCs with advancing age, and the cells become sensitive to oxidative stress (Wang et al., 2019). It leads to a ROS‐dependent disruption of cell migration and tube formation that restrict the regenerative capacity of EPCs and reduce their migration toward ischemic areas, which is crucial for repair of the damaged endothelial cells in the vasculature and reperfusion of ischemic hindlimbs of aged mice. The researchers also observed a lower secretion of VEGF and nitric oxide (NO) production in cultured *Nrf2*‐knockdown young EPCs, while induction of Nrf2 by tert‐butylhydroquinone (t‐BHQ) restores redox homeostasis and angiogenesis and suppresses NLRP3/NF‐κB pathway in aged EPCs akin to young cells. Importantly, the complex Nrf2‐mediated changes in aged EPCs are accompanied by cellular senescence, represented by increased SA‐β‐galactosidase activity and cell cycle arrest regulators p16^INK4a^ and p21^WAF1/Cip1^ (Wang et al., 2019). Not only in aging models, Nrf2 downregulation affects similar pathways regulating the redox machinery and inflammatory responses, inducing a senescence phenotype in EPCs obtained from diabetic mice (Dai et al., 2020). In diabetic EPCs, Nrf2 activation by t‐BHQ could reduce ROS levels and upregulate antioxidants SOD, HO‐1, and NQO‐1, which contribute to impaired angiogenesis (Wang et al., 2018). Moreover, increasing Nrf2 expression enhances the resistance of EPCs to oxidative damage induced by diabetes and improves the regenerative efficacy of EPCs in the mouse model of diabetic limb ischemia. This is likely achieved through the transcriptional upregulation of the rate‐limiting enzyme in the tricarboxylic acid (TCA) cycle, isocitrate dehydrogenase 2 (IDH2), which preserves mitochondrial function in diabetic EPCs (Dai et al., 2022). Dysfunctional EPCs in both patients with T2DM and diabetic mice disrupts endothelial repair and increases the risk for limb amputation, whereas Nrf2 stimulation, as a potential vascular reparative intervention, can restore the cell migration and neovascularization capacity and simultaneously rescue EPC senescence for management of diabetes mellitus. Beyond the cardiovascular system, Nrf2 is a key regulator of vascular recovery in ischemic central nervous system diseases including stroke (Wang et al., 2022) and retinopathies (Wei et al., 2015). In a mouse model of ischemic retinopathy, neuronal Nrf2 deficiency strongly suppresses vascular regrowth into retina and increases pathologic neovascularization. Wei and colleagues tested a pharmacologic activation of neuronal Nrf2 in ganglion cells to promote reparative angiogenesis and suppress pathologic neovascularization in retina. They delineated that Nrf2 activation by CDDO‐Im downregulates Sema6A, a membrane‐bound semaphorin that regulates angiogenesis, in a HIF‐1α‐dependent fashion, and thereby reprogram ischemic tissue toward neurovascular repair in avascular retina.

#### 5.1.2 In CPCs

In addition to the vasculature, Nrf2 play a key role in the repair of pathologic heart by regulating antioxidant response in CMs. Owing to insufficient CM self‐renewal and regenerative capacity during aging and cardiac pathologies, an efficient strategy for reactivating primitive renewal and reparative abilities may promote the mature mammalian heart repair. Accordingly, for instance, Martin and his team reported that *Paired‐like homeodomain 2* (*Pitx2*) is crucial for repair in neonatal hearts; hence *Pitx2* gain‐of‐function induced the reparative capacity in injured ventricular cardiomyocytes after myocardial infarction (MI). Pitx2 is a downstream target of Nrf2 that regulates a subset of genes encoding ROS scavengers by activating the Hippo‐Yap pathway, which is essential for postnatal cardiac regeneration, to preserve redox balance in the adult myocardium (Hill et al., 2019; Tao et al., 2016). They revealed that the Nrf2‐Pitx2 genetic pathway is activated by cardiac damage and promotes cardiac repair by enhancing antioxidant response. Consistently, the activation of Nrf2 by *intraperitoneal* injection of a multifaceted fluorinated compound TT‐10 (C_11_H_10_FN_3_OS_2_) could induce Yap nuclear translocation and Wnt/β‐catenin signaling, by which increased CM proliferation ameliorated cardiac remodeling and diastolic dysfunction in a mouse model of MI (Hara et al., 2018). Not only in an in vivo model, but in cultured CMs, TT‐10 upregulates Nrf2 target genes encoding antioxidants *Txn1* and *Hmox1*. It is worth noting that due to the potential of Nrf2 activation in cardiac regeneration, several compounds targeting Nrf2 have been tested for their ability to promote cardiac regeneration following myocardial injury. Another such compound is the cannabinoid receptor type 2 (CB2) agonist AM1241, which has been observed to activate protein kinases PI3K/Akt‐Nrf2 and increase the expression of antioxidant enzyme HO‐1 in infarcted myocardium (Li et al., 2016; Wang et al., 2014). These mechanisms converted the cardiac postischemic microenvironment to a favorable microenvironment for proliferation of resident CPCs and thereby enhanced endogenous cardiac tissue repair after MI in mice. As discussed, Nrf2 has been a common target in a number of studies investigating myocardial regeneration following MI in animal models.

The existing evidences on the role of Nrf2 in stem cell self‐renewal and proliferation may explain the failure to maintain stem/progenitor cell populations and impaired ability of these cells to regenerate cardiac and vascular tissues in premature and physiological aging. Consistently, the repression of Nrf2 disrupts antioxidant defense system in both differentiated and stem cells in the vasculature of HGPS patients with premature aging phenotype (Atchison et al., 2020; Kubben et al., 2016; Villa‐Bellosta, 2020). These patients recapitulate many symptoms of physiological vascular aging, mainly severe arterial stiffening, fibrosis, and generalized atherosclerosis, which are recognized to underlie AAD, as the “vascular theory of aging” declares (Gkaliagkousi et al., 2022; Le Couteur & Lakatta, 2010). Progerin, a mutated form of prelamin A variant that induces cellular senescence (Aguado et al., 2019), is accumulated in their stem cells that impairs cellular maintenance and differentiation by inducing the expression of genes related to oxidative stress and the endoplasmic reticulum (ER) stress responses and the related unfolded protein response (Harhouri et al., 2017). This protein is also found in CMs and in vascular cells (EC and VSMC) of *Lmna* ^*LCS/LCS*^ progeria mice that exhibit adventitial thickening and aortic calcification (Hamczyk et al., 2018). Suppressed Nrf2 activity alongside by increased oxidative stress is sufficient to recapitulate premature aging defects (Kubben et al., 2016). Yet, the upregulation or translocation of Nrf2 to the cell nucleus facilitates the transcription of downstream antioxidant genes necessary for the regulation of stress responses, metabolism, and pluripotency in cardiac and vascular stem cells, and improves overall health and lifespan in these animals (Dai et al., 2020). Therefore, targeting Nrf2 emerges a therapeutic strategy for revascularization and vascular repair in AAD, namely diabetes, and protecting against cardiovascular stem/progenitor cell exhaustion and aging (Rampin et al., 2022; Wang et al., 2018, 2019). However, much remain to fully understand whether and how Nrf2 regulates the regenerative mechanisms involved and the Nrf2‐targeted therapies are effective in aging‐related CVD in old humans.

### 5.2 HIF‐1α

#### 5.2.1 In EPCs

HIF‐1α has been demonstrated to regulate pathways controlling EPC mobilization and endothelial differentiation (Bosch‐Marce et al., 2007). Hypoxic conditions stimulate HIF‐1α activation in EPCs, which in turn promotes the secretion of angiogenic factors, such as VEGF and stromal cell‐derived factor 1 (SDF‐1). HIF‐1α‐mediated upregulation of VEGF and SDF‐1 enhances EPC recruitment to injured sites and facilitates neovascularization (Lee et al., 2013). With advancing age, HIF‐1α expression and activity decline, leading to reduced EPC function and impaired angiogenic capacity (Chang et al., 2007; Kim et al., 2011). It has been demonstrated long time ago wild‐type old mice are afflicted with inadequate perfusion recovery in ischemic limb accompanied by transcriptional alterations in hypoxia‐related genes encoding angiogenic cytokines ANGPT1, ANGPT2, MCP‐1, PLGF, SCF, SDF‐1, and VEGF in calf muscles to levels similar to those in heterozygous *Hif1a*‐knockout mice (Bosch‐Marce et al., 2007). In these mice, adenovirus (AdCA5)‐based HIF‐1α gene therapy improved limb perfusion in older mice and increased the number and mobilization of circulating angiogenic cells in both nonischemic and ischemic limbs in old mice. Consistently, activation of HIF‐1α‐TWIST axis by hypoxia preconditioning tightly downregulates p21^WAF1/Cip1^ in cultured senescent EPCs, and thereby rescues senescence and improves cellular proliferation and survival (Lee et al., 2013). In addition to EPCs, HIF‐1α, intriguingly, underpins the activation of the transcription factor *Etv2* in ESCs followed by Notch1 signaling, necessary for redox homeostasis and expression of vasculaoprotective genes such as *NOS3*, *NNT*, *GRX*, and PTGS1 in generated arterial ECs (Tsang et al., 2017). Tsang and colleagues proved a successful outcome of transplantation of functional arterial ECs transitioned from EPCs, in which sustained activation of HIF‐1α was sustainably activated, in mouse myocardial and hindlimb ischemia models. Accordingly, ECs derived under hypoxia as opposed to normoxia showed engraftment in ischemic tissues, thus leading to local revascularization and restored cardiac function (Alique et al., 2019; Yang et al., 2017). Despite the considerable attention HIF‐1α has attracted as a target for revascularization in peripheral ischemic tissues, there is a limited knowledge on the effects of HIF‐1α‐based therapies in atherosclerotic cardiovascular diseases in aging.

#### 5.2.2 In CPCs

Beyond, hypoxic niche in adult heart, in particular mammalian epicardium and subepicardium, provides a safe environment for adult CPCs and regulates metabolic pathway in a favor of glycolysis to preserve cardiac homeostasis and regenerative capacity (Kimura & Sadek, 2012; Korski et al., 2019). These layers have the lowest capillary density, where over 50% of CMs express HIF‐1α protein. HIF‐1α has been identified as a master regulator of hypoxic responses that bridges transcriptional alterations and metabolic reprogramming toward a reductive state to counteract oxidative stress‐induced senescence and exhaustion in these cells (Alique et al., 2020; Bellio et al., 2016). Accordingly, in vitro studies revealed that hypoxia preconditioning promotes murine CSC survival and cardiogenic differentiation (represented by increased α‐SA and cTnT expression) in a HIF‐1α/apelin/APJ‐mediated fashion (Hou et al., 2017). Under hypoxic conditions, HIF‐1α stabilizes and activates various downstream targets involved in CPC maintenance and function, including *Oct4*, *Nanog*, and *GATA4*, the genes which classically involved in cardiac development during embryogenesis and in cardiac reprogramming and regeneration in adult heart. Activation of HIF‐1α in CPCs promotes angiogenesis, inhibits apoptosis, and enhances their regenerative capacity. These may drive cardiac tissue turnover during normal aging and post‐myocardial injury (Sayed et al., 2022). However, dysregulation of HIF‐1α signaling in aging impairs the responsiveness of CPCs to hypoxia, leading to compromised cardiac repair mechanisms. Multiple factors contribute to the decline in HIF‐1α expression and activity during aging (Röning et al., 2022). Accumulating evidence suggests that age‐related changes in the progenitor/stem cell microenvironment, including oxidative stress, chronic inflammation, and cellular senescence, impair HIF‐1α signaling (Marino et al., 2022; Sweeney et al., 2022). Dysfunctional mitochondria, decreased oxygen availability, and altered epigenetic regulation also contribute to age‐related decline of HIF‐1α in CPCs (Gevaert et al., 2017; Zhang et al., 2007). Understanding these mechanisms is crucial for developing strategies to preserve or restore HIF‐1α function and rejuvenate the regenerative capacity of progenitor/stem cells in the aging cardiovascular system.

## 6 REDOX HOMEOSTASIS: VITAL FOR CELL REPROGRAMMING TO CARDIOVASCULAR LINEAGES

**FIGURE 4:**
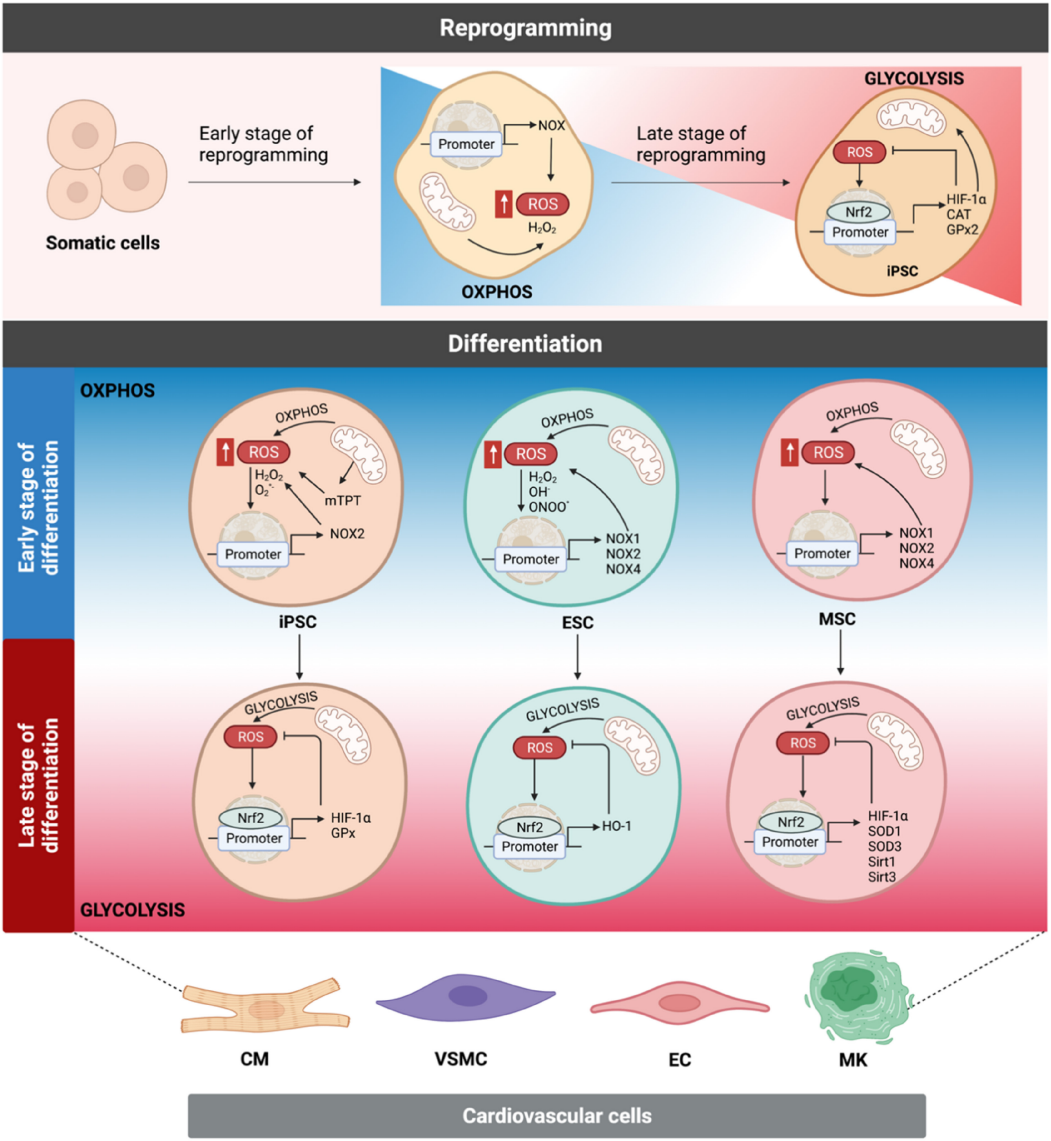
Redox homeostasis in cardiovascular reprogramming. (upper) At early stage of iPSC reprogramming, forced induction of specific pluripotency transcription factors [OCT4, SOX2, KLF4, and c‐MYC (OSKM)] in somatic cells fosters iPSC generation where essential H_2_O_2_ increase is accompanied by oxidative phosphorylation (OXPHOS). Nrf2 senses ROS and activates cytoprotective antioxidants HIF‐1α, Gpx2, and CAT, which reduce intracellular ROS and thereby shift metabolism toward glycolytic energy production at the later stage of cell reprogramming. (lower) In iPSCs and ESCs, NOX family or mitochondrial permeability transition pore (mPTP) opening contribute to intracellular ROS accumulation and OXPHOS at the onset stage of CV lineage differentiation. These induce Nrf2 upregulation of antioxidant (e.g., HIF‐1α, HO‐1, and SIRT1 and 3) and scavenging (e.g., SOD1, SOD3, and Gpx) machinery genes and glycolytic shift in order to counteract a transient oxidative cell cycle arrest. Nrf2‐regulated redox homeostasis triggers the later stage of differentiation. Images created with BioRender.com.

The recent investigations by high‐throughput NGS‐RNA sequencing of human iPSC and iPSC‐derived cardiovascular cells appreciate the complexity and importance of redox regulation during cell reprogramming (Dudek et al., 2021; Nugud et al., 2018). Thus, targeting redox signaling in iPSCs may enhance its efficiency for cell‐based therapy (Figure 4). As discussed earlier in this review, ROS confer differential effects on stem cell biology (Saeedi Saravi et al., 2020). In the recent years, in vitro iPSC studies revealed that H_2_O_2_ elevation is pivotal to induce pluripotency at the time of cell reprogramming (Sinenko et al., 2021; Tatapudy et al., 2017). Accordingly, there are a few studies that demonstrate an increased intracellular H_2_O_2_ generation, particularly in the cell mitochondria, at early stages of forced cell reprogramming, leads to the onset of nuclear reprogramming (Lee et al., 2012; Zhou et al., 2016). In one study, Cooke and colleagues subjected mouse embryonic fibroblasts (MEFs) to retroviral vectors carrying genes encoding *Yamanaka* transcription factors (*Oct4*, *Klf4*, *Sox2*, and *c‐Myc*) for forced iPSC reprogramming. They showed that depletion of H_2_O_2_ by antioxidants or NOX inhibitors at the early phase can decrease reprogramming efficiency (Zhou et al., 2016). Moreover, in 3 T3‐L1 white pre‐adipocytes, silencing *p22* ^*phox*^—a critical subunit of the NOX 1–4 complexes—or addition of selective ROS scavengers EUK134, Ebselen and Mito‐TEMPO reduced iPSC reprogramming efficiency (Zhou et al., 2016). Consistently, elevation of ROS generation in mitochondria by transient opening of mitochondrial permeability transition pore (mPTP) at the early phase optimizes somatic cell reprogramming both metabolically and immunologically (Meng et al., 2018; Trajano & Smart, 2021; Ying et al., 2018). Nevertheless, the emphasis in these studies is on critical role of redox homeostasis in both early and late phases of reprogramming, as inferred by an impaired pluripotency and cellular damage resulted from excessive ROS exposure.

Upon reprogramming of somatic cells into iPSC, at the differentiation stage, ROS contributes to iPSC redifferentiation to target cells by regulating mitochondrial metabolism and its oxidative state (Wang et al., 2013). For instance, similar to what seen in the iPSC reprogramming, ROS generation exceeds by the NOX enzymes or the mitochondrial electron transport chain at the early phase of iPSC‐to‐CM differentiation. Consistently, mPTP‐mediated activation of mitochondrial oxidative metabolism plays a key role in the differentiation process (Garbern & Lee, 2021). In addition to CM differentiation, stimulation of NOX2 has been observed to boost the differentiation efficiency of iPSC to ECs through activation of Notch signaling pathway. Accordingly, *Nox2* ^*−/−*^ mouse iPSCs (miPSCs) exhibit a decreased expression of arterial endothelial markers ephrinB2, neuropilin 1 (Nrp1), and activin receptor‐like kinase 1, which has been accompanied by upregulated Notch components, in particular Notch1 (Kang et al., 2016). In these cells, NOX2‐knockout, despite the presence of an effective differentiation cocktail (including β‐mercaptoethanol, BMP‐4, VEGF, and nonessential amino acids), drastically reduces ROS levels and thereby disrupts cell survival and angiogenic potency of miPSC‐derived ECs. In a mouse model of hind‐limb ischemia, the mice receiving *Nox2* ^*−/−*^‐miPSC‐ECs showed a markedly lower capillary and arterial density in the ischemic limbs, as opposed to mice transplanted with wild‐type miPSC‐ECs. This study hints the importance of oxidants, in a temporal fashion, for effective iPSC‐EC differentiation and subsequent vascular repair (Maraldi et al., 2021).

According to the abovementioned examples, in the process of differentiating iPSCs into both CMs and ECs, the upregulation of ROS production during the early phase is of critical importance (Dai et al., 2020; Gong et al., 2021; Jahng et al., 2021). However, excessive ROS levels trigger the activation of antioxidant regulators such as Nrf2 and HIF‐1α, along with inducing changes in mitochondrial structure and function (Dai et al., 2020). These adaptations facilitate a metabolic shift from oxidative phosphorylation to glycolysis, which serves as a protective mechanism for the cells during the later phase of differentiation (Hawkins et al., 2016). Thus, the antioxidant transcription factors are vital for governing the cellular redox balance and a conductive microenvironment necessary for successful differentiation of iPSCs into specialized cardiac and endothelial cell lineages. It highlights their significance in enhancing the overall efficiency and fidelity of the iPSC differentiation process. In order to elucidate the significant contribution of Nrf2 in cell reprogramming, Nrf2‐knockout (*Nfe2l2* ^*−/−*^) in human fibroblasts led to an elevated ROS production and a decreased expression level of HO‐1 (Stepniewski et al., 2018). Consequently, this reduction in Nrf2 function resulted in a compromised pluripotency and differentiation capacity of the fibroblasts. Moreover, in *Hmox1* ^*−/−*^ iPSCs, the differentiation process toward contracting CMs expressing myocardin and *GATA4* was also impaired, leading to attenuated cardiac differentiation. Similarly, CRISPR/Cas‐9‐mediated *Hmox1* silencing in murine ESCs impairs their spontaneous cardiac differentiation. Yet, Nrf2 activation with sulforaphane or HO‐1 induction with CoPPIX in *Oct4*‐transfected MEFs induces nuclear import of Nrf2, which increases the expression of HO‐1 and further iPSC colonies. These studies demonstrate that genetic deletion of either *Nfe2l2* or *Hmox1* in both iPSCs and human fibroblasts elevates cellular levels of p53 and p53‐regulated miR‐34a, which arrest cell cycle and thereby induce cellular senescence (Choi et al., 2017; Stepniewski et al., 2018). The findings are intriguingly in line with the effects of H_2_O_2_ at lower concentrations, which elicit a robust upregulation of the p53 isoform *Δ133p53* in CMs at the wound site of ventricles in zebrafish. This upregulation of *Δ133p53* is shown to play a crucial role in promoting heart regeneration (Ye et al., 2020). Mechanistically, this phenomenon can be attributed to the compensatory expression of antioxidant and proliferative genes in response to short‐term H_2_O_2_ generation. The p53 target genes, including antioxidants *gpx1a*, *sesn2*, *aldh4*, *sesn1*, *sod1*, *sod2*, and the HIF‐1α signaling pathway (*hif1al2* and its downstream genes *jak2a* and *pim2*) are responsible for maintaining redox homeostasis within the regenerating tissue (Harhouri et al., 2017; Nakamura et al., 2021). Using cutting edge single‐nucleus RNA sequencing of regenerating neonatal CMs, Cui and her colleagues observed the involvement of stress‐responsive transcription factor Nrf1 (Cui et al., 2020). As a member of the Nrf family, Nrf1 binds to the endoplasmic reticulum and plays a significant role in co‐regulating cardioprotection and heart regeneration during stress induced by I/R injury in the adult mouse heart. Nrf1 achieves this by simultaneously activating HO‐1 and a regenerative program that involves genes associated with proteasome‐mediated proteolysis. Furthermore, Nrf1 has been found to regulate adaptive stress responses in hiPSC‐CMs. It acts to safeguard these cells from doxorubicin‐induced cardiotoxicity while also activating a transcriptional program essential for CM regeneration, thereby maintaining tissue homeostatic balance (Cui et al., 2021).

In line with the temporal role of oxidants in promoting cell reprogramming, emerging consensus underscores the importance of “time‐specific” and targeted antioxidant interventions to mitigate oxidative stress, prevent cellular damage, and facilitate the reprogramming and redifferentiation of iPSCs as well as the functional integration of newly regenerated cardiovascular cells. For instance, vitamin C and selenium have demonstrated increased efficiency in direct reprogramming of mouse fibroblasts into beating CMs (Shafi et al., 2019; Suzuki & Shults, 2019); however, whether this effect is linked to redox regulation remains uncertain. Vitamin C's impact might be attributed to its epigenetic regulation of pluripotency machinery, involving the induction of jumonji‐domain containing histone demethylase *Jhdm1a/1b*‐mediated H3K36me2/3 demethylation (Wang et al., 2011). Despite the beneficial effects observed in antioxidant intervention during direct and forced reprogramming, controversies exist in forced cell reprogramming. Notably, the administration of a subset of antioxidants, such as vitamins B1 and E, sodium selenite, N‐acetyl‐L‐cysteine (NAC), resveratrol, α‐lipoic acid, and L‐carnitine, did not replicate the effects of vitamin C in facilitating *Sox2*/*Klf4*/*Oct4*‐mediated forced reprogramming process in mouse and human fibroblasts (Esteban et al., 2010; Shults et al., 2019; Suzuki & Shults, 2019).

These findings collectively underscore the intricate and specific roles of oxidants and antioxidants in reprogramming strategies, emphasizing the necessity for a precise and individualized approach to harness their benefits for effective cell reprogramming and cardiovascular regenerative therapies. Our conclusion highlights the significance of both ROS and antioxidants at specific time points during somatic cell reprogramming into iPSCs and their subsequent differentiation into functional target cells. However, it should be noted that excessive and sustained ROS levels may lead to stem/progenitor cell senescence and impair their survival and renewal capacity. Furthermore, the concept of using ROS stimulants in cell reprogramming and regenerative strategies in clinical models remains unproven. Nevertheless, in vitro studies using cultured iPSCs have revealed the detrimental effects of deleting ROS‐generating enzymes on reprogramming efficiency. In contrast, a limited number of animal and clinical investigations demonstrate that a time‐ and concentration‐dependent approach involving antioxidants or upregulation of antioxidant genes, as well as innovative pharmacological cell‐based strategies, is essential for optimizing cellular reprogramming. Enhancing antioxidant defense mechanisms may effectively improve the renewal and proliferation capacity of tissue‐resident stem/progenitor cells, fostering cardiac and vascular regeneration and repair within the body.

## 7 CHEMICAL ENTITIES TARGETING REDOX SYSTEM FOR CARDIOVASCULAR REGENERATION

**FIGURE 5:**
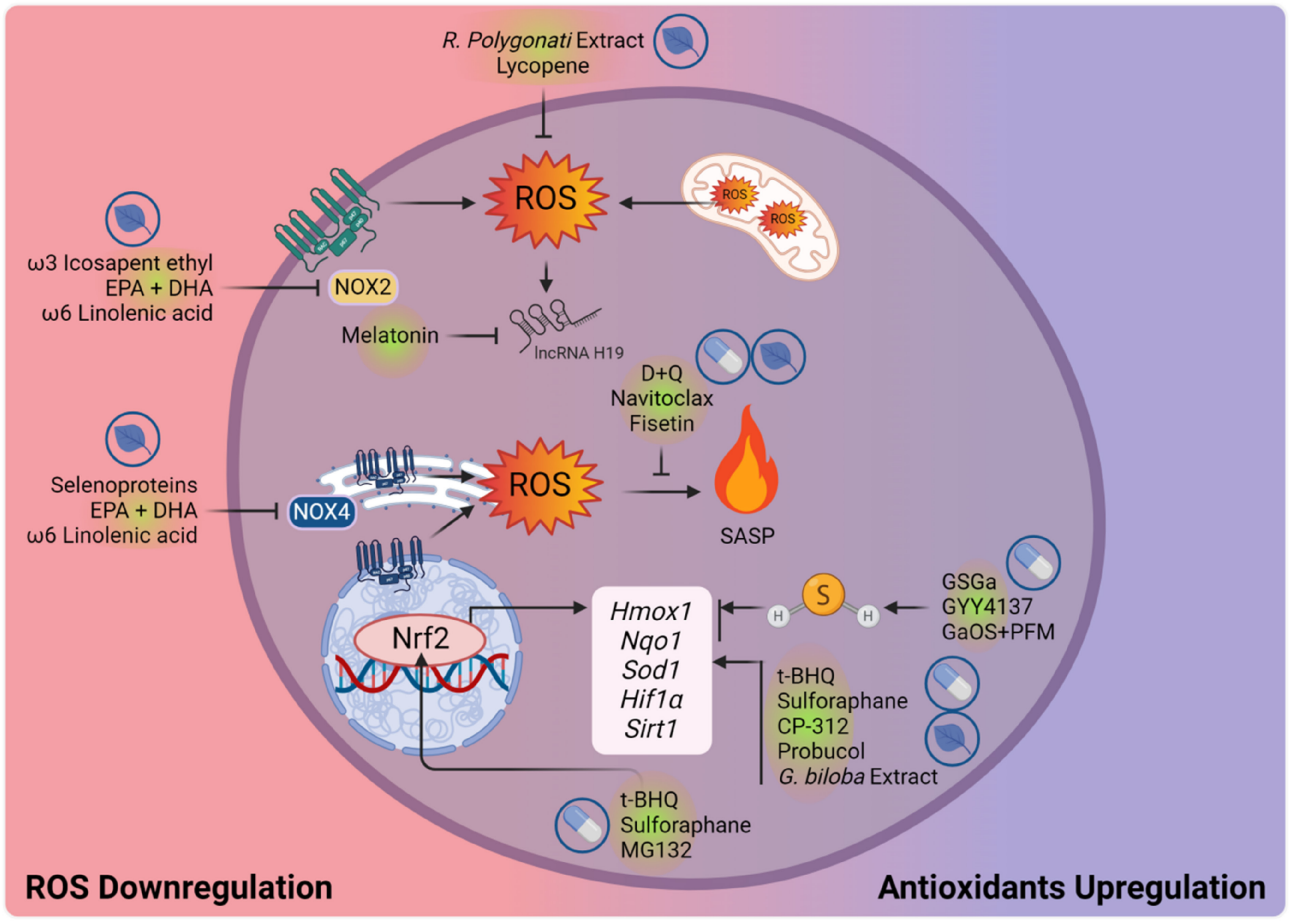
Schematic representation of bioactive chemical compounds with potential action for regulation of redox machinery in CPCs and EPCs, leading to increased regenerative capacity. It depicts the mechanisms of natural/synthetic compounds (left side) downregulating ROS generation or (right side) upregulating antioxidant genes/molecules (H_2_S). Images created with BioRender.com.

Over the past decade, a multitude of redox regulators has been investigated for their potential in cardiovascular regeneration (Ballard & Edelberg, 2007; Elhelaly et al., 2016). However, only a limited number of these regulators have progressed to clinical application. Several pharmacological redox regulators, both natural and synthetic, have been identified to directly or indirectly protect cardiovascular stem/progenitor cells and optimize cell‐based cardiovascular regenerative therapies. Many of these redox‐modulating small molecules function by inducing Nrf2 and downstream antioxidant enzymes, particularly HO‐1, to regulate the oxidant–antioxidant interplay in cardiovascular stem/progenitor cells and pluripotent stem cells crucial for generating cardiovascular lineages (Loboda et al., 2016; Wu et al., 2022). Additionally, these compounds inhibit oxidative pathways by downregulating the NOX family (Zhang, Murugesan, et al., 2020; Zhang, Zhang, et al., 2020) or enhance antioxidant defense (Shafi et al., 2019; Suzuki & Shults, 2019) by upregulating sirtuins (Xie et al., 2023) or releasing hydrogen sulfide (Zhang et al., 2018) in the cells. Moreover, an emerging strategy involves senolytic therapies, which aim to rejuvenate the regenerative capacity of aged cardiovascular stem/progenitor cells, thereby fostering a more efficient regenerative approach (Owens et al., 2021; Robbins et al., 2021; Zhu et al., 2015). In this context, we focus on redox‐targeting small molecules or natural compounds that have been identified for their protective effects on cardiovascular stem/progenitor cells under injury‐induced stress and their ability to improve regenerative capacity during aging (see Figure 5 and Table 1 for a summary). These advances in redox modulation hold promise for enhancing cardiovascular regenerative strategies and may pave the way for future therapeutic interventions.

**TABLE 1:** Redox‐modulating compounds capable of cardiovascular regeneration in experimental and clinical models.

Compound	Dose and schedule	Species	Mechanism of action	Outcome	Ref.
**Nrf2 inducers**					
t‐BHQ	10–80 μM, for 6–24 h	Mouse; EPC	Nrf2 activation Upregulation of redox regulators (Nrf2, HO‐1, NQO1, SOD) and NO Decrease in MDA levels	Rescues EPC senescence	Wang et al. (2018)
Sulforaphane	1 and 5 μM, for 4 h	Human; ECFC	Nrf2 activation Upregulation of HO‐1 Reduction of intracellular H_2_O_2_	Preserves endothelial sprouting and tube formation	Gremmels et al. (2017)
MG132	0.15–10 M, for 24–48 h (cells); 1 and 10 μg/kg (mice)	Human; iPSC‐VSMC; *Lmna* ^*G609G/G609G*^ mice	Nrf2 activation Downregulation of SRSF‐1 and ‐5	Increases progerin clearance	Harhouri et al. (2017)
**HO‐1 inducers**					
CP‐312	10 μM, for 2 h	Human; iPSC‐CM	HO‐1 induction Upregulation of Nrf2/HMOX1, NQO1	Protects hiPSC‐CMs against oxidative stress	Kirby et al. (2018)
Probucol	500 mg/kg/day, for 3 days prior to & 1 month with PM_2.5_ exposure; oral gavage	Mouse; exposed to ambient air containing PM_2.5_	Inhibition of intracellular ROS production and pro‐inflammatory cytokines TNF‐α, IL‐1α, and IL‐6	Protects EPCs from PM_2.5_ damage	Chen et al. (2018)
Probucol	0.75% w/w, for 6 weeks	New Zealand white rabbit; model of balloon‐damaged arteries (2% w/w cholesterol‐enriched diet)	Activation of NO‐cGMP pathway Suppression of oxidative events	Stimulates endothelial regeneration Improves re‐endothelialization Inhibits intimal thickening	Lau et al. (2003)
*Ginkgo biloba* extract	100 or 300 mg/kg/day for 14 days before injury and 28 days after injury	Mouse model of femoral artery injury	HO‐1 induction Activation of PI3K/Akt/eNOS signaling (in human late EPCs) and of p38 pathway (in SMPCs)	Activates early & late EPCs Suppresses the migration of SMPCs Improves in vivo vascular repair	Wu et al. (2019)
**H**_**2**_ **S‐releasing compounds**					
NaHS	25–500 μM (preconditioning)	Mouse model of carotid artery injury; Human peripheral blood EPC	H_2_S release Upregulation of AMPK/eNOS	Increases the capacity of re‐endothelialization & vascular repair	Zhang et al. (2018)
GaOS + PFM	4.2 and 25.2 μg, for 3 days	Human; CSC	H_2_S release	Enhances the growth of CSC in the presence of H_2_O_2_‐induced oxidative stress	Cacciotti et al. (2018)
GSGa or GYY4137	5‐20 μL/mL (GSGa), 25–200 μM (GYY4137)	Human; Sca1^+^ Lin^−^ CSC	H_2_S release Increased expression of HIF‐1α and NQO1	Enhances the proliferation and migration of CSC in the presence of H_2_O_2_	Di Giovanni et al. (2020)
**Statins**					
Simvastatin nanoparticles	Simvastatin nanoparticle‐loaded hAdSCs	Mouse model of MI (LAD ligation); hAdSCs		Increases stem cell proliferation and migration Increases hAdSC differentiation Upregulates *Nkx2.5* and *GATA4* transcription Reduction in cardiac fibrotic area Improves cardiac function	Yokoyama et al. (2019)
Rosuvastatin or Simvastatin or Pravastatin	1 μM or 5 μM, for 24 h	Mouse and Rat; CSC	Akt activation	Enhances CSC survival Protects from H_2_O_2_‐induced apoptosis and cell death	Nakashima et al. (2018)
Rosuvastatin	20 mg/kg/day in drinking water, for 14 days before and 28 days after LAD ligation	Rat model of MI	Increases in NO bioavailability and Akt phosphorylation	Increases cardiomyocyte differentiation Commitment of c‐kit^+^/GATA4^+^ CSC in injury site	Cianflone, Torella, et al. (2020), Cianflone, Cappetta, et al. (2020)
Atorvastatin	50 mg/kg/day by gavage, for 4 weeks starting on day 1 after MI	eNOS^−/−^ mice; Mouse model of MI	Increase in NO bioavailability	Improves left ventricular dysfunction Reduces cardiac interstitial fibrosis Enhances animal survival	Landmesser et al. (2004)
**Natural compounds**					
Bavachalcone	1 & 2 μM, for 14 days	Mouse model of hind‐limb ischemia; BM‐EPC	Increase in AMPK activity & the expression of MnSOD Activation of RORα–EPO–AMPK axis	Enhances EPC differentiation Increases ischemic hind‐limb blood flow, circulating EPCs, and capillary angiogenesis	Ling et al. (2017)
Lycopene	10–50 μg/mL, for 24–72 h	Rat; BM‐derived EPC	Suppression of AGE‐induced oxidative autophagy	Increases the proliferation of EPC in DM rats	Zeng et al. (2017)
Visfatin	0.1–10 nM, for 30 min	Human; BM‐derived EPC	Reduction in ROS generation Increases telomerase activity Upregulation of SIRT1 Downregulation of p53	Rescues oxLDL‐induced cellular senescence Increases EPC proliferation Increases tube formation	Ming et al. (2016)
*Angelica sinensis* polysaccharides	20–100 μg/mL, for 48 h	Mouse; BM‐derived EPC	Decrease in gp91‐phox expression Reduction in oxidative stress Increases telomerase activity Increase in Akt/hTERT phosphorylation	Rescues oxLDL‐induced cellular senescence Increases EPC proliferation	Lai and Liu (2015)
*Rhizoma Polygonati* extract		Rat; BM‐derived EPC	Reduction in ROS generation	Rescues cellular senescence Increases EPC proliferation and angiogenesis in aged rats	Qin (2019)
Melatonin	10 nM‐100 μM, for 2 h	Mouse; c‐kit^+^ CPC	Upregulation of H_2_O_2_‐induced downregulated LncRNA H19 H19/miR‐675/USP10 pathway	Blocks premature senescence of CPC in response to oxidative stress	Ma et al. (2018)
Melatonin	100 μM	Mouse; ESC	Upregulation of *MYH6* & *7* Reduction in *HIF1A* transcription and stabilization Enhancement of SIRT1 levels	Enhances cardiomyogenesis from mouse ESC	Hardeland (2019)
Selenium	20 or 50 ng/mL, for 15 days	ESC	Reduction in NOX4 activity Upregulation of thioredoxin	Facilitates the differentiation of ESC‐to‐vascular progenitor cells	Tinkov et al. (2020)
Icosapent ethyl	2 × 1 g, twice daily for 3 months	Human (IPE‐PREVENTION trial; NCT04562467)	Suppression of oxidative stress	Ongoing; Lowering depletion and dysfunction of circulating EPC (CD34^+^CD133^+^VEGFR2^+^ cells)	National Library of Medicine (2020)
EPA + DHA + ascorbic acid	25 + 25 + 100 μM, for 48 h	Rat model of MI; ESC‐derived cardiac lineage	Upregulation of HO‐1, and cardiac markers (*GATA4* and *MYH6*) Downregulation of fibroblast markers (vimentin and Col1)	Increases cell viability against H_2_O_2_‐induced oxidative stress Reduces fibrosis in the ischemic myocardium after transplantation	Shabani et al. (2019)
EPA or Linoleic acid	1–100 μM, for 3–10 days	Mouse; ESC	Increase in NOX‐mediated ROS production, eNOS phosphorylation, & NO bioavailability Upregulation of energy sensors AMPK and PPAR‐α	Enhances vascular differentiation of ESC via increased vascular structure formation and expression of the endothelial‐specific markers CD31 and VE‐cadherin	Taha et al. (2020)
**Senolytics**					
Dasatinib + Quercetin	5 mg/kg Dasatinib +50 mg/kg Quercetin, for 3 consecutive days every 2 weeks for 2 months	Human; CPC and Aged mice (INK‐ATTAC); CPC	Suppression of SASP inflammatory and oxidative responses Inhibition of telomere shortening Inhibition of BCL‐2 family	Activates resident CPCs in vivo Eliminates senescent CPCs, increases the number of small Ki67^+^ CMs Regenerates or restores cardiac function	Lewis‐McDougall et al. (2019), Zhu et al. (2015)
Navitoclax or Fisetin	0.5–20 μM				
Dasatinib + Quercetin	5 mg/kg Dasatinib +50 mg/kg Quercetin, *intraperitoneal* injection, for 3 consecutive every week for 4 weeks	Human and Mice (diabetic); CPC	Suppression of ROS generation Suppression of SASP Inhibition of SCAPs Downregulation of p16^INK4a^	Rescues CPC senescence Restores CPC proliferation, myogenic differentiation, and regeneration Improves myocardial remodeling Improves cardiac diastolic function	Marino et al. (2022)
**Biomaterials**					
Melanin/Alginate hydrogels	0.2, 0.5, 1.0, 2.0 mg/mL, injection in the infarct area (100 μL)	Rat model of MI	ROS scavenging Promotion of macrophage polarization to regenerative M2 phenotype Suppression of inflammatory and apoptotic responses	Protects against oxidative stress‐induced CM injury Increases angiogenesis Restores cardiac structure and function post‐MI Improves cardiac repair	Zhou et al. (2021)
Graphene oxide/Alginate microgels	2.0 mg/mL, injection in the infarct area	Mouse model of MI	Suppression of ROS generation Promotion of macrophage polarization to regenerative M2 phenotype Suppression of inflammatory response	Increases ESC survival in H_2_O_2_ microenvironment Promotes CM differentiation Restores cardiac structure and function Improves cardiac repair	Choe et al. (2019), Han, Kim, et al. (2018), Han, Bedarida, et al. (2018)
Fullerenol/Alginate hydrogel	Implanting fullerenol/alginate hydrogel‐BADSCs in the infarct area	Rat model of MI	Suppression of oxidative stress Activation of ERK and p38‐regulated pathways Inhibition of JNK pathway	Promotes BADSC‐CM differentiation Improves cardiac repair	Hao et al. (2017)
Polyurethane fibrous patches	Implanting polyurethane patch in the infarct area	Rat model of MI	Potentiates antioxidant defense	Protects from CM death Improves the reconstruction of cardiac function Enhances revascularization in the infarct area	Yao et al. (2020)

Abbreviations: Akt, protein kinase‐B; AMPK, AMP‐activated protein kinase; BADSC, brown adipose‐derived stem cells; CAD, coronary artery disease; Col1, collagen type 1; CPC, cardiac progenitor cell; DHA, docosahexaenoic acid; ECFC, endothelial colony‐forming cell; eNOS, endothelial nitric oxide synthase; EPA, eicosapentanoic acid; EPC, endothelial progenitor cell; ERK, extracellular signal‐regulated kinase; ESC, embryonic stem cell; GaOS + PFM, garlic oil‐derived H_2_S‐releasing poly(lactic) acid fibrous membranes; GSGa, GSH–garlic conjugates; H_2_O_2_, hydrogen peroxide; H_2_S, hydrogen sulfide; HGPS, Hutchinson–Gilford progeria syndrome; HIF‐1α, hypoxia‐inducible factor‐1α; HO‐1, heme oxygenase‐1; JNK, c‐Jun N‐terminal kinase; MI, myocardial infarction; NaHS, sodium hydrosulfide; NO, nitric oxide; NOX, NADPH oxidase; NQO1, quinone oxidoreductase‐1; Nrf2, nuclear factor erythroid 2‐related factor 2; PPAR, peroxisome proliferator‐activated receptor; SDF‐1, stromal cell‐derived factor‐1; SOD, superoxide dismutase; SRSF‐1, serine and arginine‐rich splicing factor‐1; t‐BHQ, *tert*‐butylhydroquinone.

This review primarily focuses on the beneficial implications of redox‐targeting molecules and antioxidants concerning the protection and regeneration of cardiovascular stem/progenitor cells. However, a contentious debate surrounds the neutral or negative outcomes observed in clinical trials involving natural or synthetic antioxidants for vascular regeneration. Over the past two decades, meta‐analyses of randomized controlled trials, involving hundreds of thousands of human subjects, have uncovered the failure of synthetic antioxidant supplements in achieving vascular regeneration and preventing major cardiovascular adverse events and mortality (Leopold, 2015; Yang et al., 2022; Ye et al., 2013). These failures are often attributed to the administration of high doses, toxicity, and insufficient short‐term supplementation. In contrast, smaller clinical trials investigating natural antioxidants, such as vitamins A, C, D, and E, and β‐carotene, have demonstrated partial success in cardiovascular cell reprogramming, especially in the context of vascular regeneration during aging (Shafi et al., 2019). Additionally, promising outcomes have been observed with mitochondria‐targeted antioxidant (MitoQ), which comprises ubiquinol fused to a lipophilic cation (Rossman et al., 2018). MitoQ has shown high efficacy for vascular regeneration by promoting endothelial cell proliferation while concurrently downregulating oxidant‐regulated cell‐cycle arrest and senescence processes in both aged humans and mice (Gioscia‐Ryan et al., 2014; Huang et al., 2022; Masoumi‐Ardakani et al., 2022). Despite these encouraging findings, challenges persist in establishing efficient and potent antioxidant‐containing regimens for cardiovascular regeneration, particularly when it comes to conducting large‐scale trials. Further research and systematic exploration are necessary to overcome these challenges and advance the development of effective antioxidant‐based therapies for cardiovascular regeneration.

### 7.1 Nrf2 inducers

Nrf2 inducers appear as promising strategy to protect EPCs against the conditions which lead to oxidative stress‐induced senescence. Sulforaphane decreases intracellular ROS in human ECFCs and preserves angiogenesis (Gremmels et al., 2017). In mouse EPC, preconditioning with another Nrf2 inducer, t‐BHQ, upregulates antioxidant genes and reduces ROS, by which protects the cells from cellular senescence (Wang et al., 2018). MG132 is a proteasome inhibitor that strongly activates Nrf2 and thereby both induces the degradation and reduces the production of progerin, a truncated and toxic prelamin A protein which is improperly processed in HGPS cells nuclei and is a hallmark of the premature aging, in human and mouse HGPS iPSC‐VSMC. This small‐molecule has a potential to protect against premature‐aging cardiovascular pathophysiology (Harhouri et al., 2017).

### 7.2 HO‐1 inducers

Pharmacological preconditioning is considered as an effective strategy to stimulate stem cell survival. Recent development of phenotypic chemical biology screening approach suggested cardioprotectant‐312 (CP‐312) as a novel small‐molecule HO‐1 inducer that protects CMs derived from hiPSC against oxidative stress (Kirby et al., 2018). These are consistent with previous data that showed protective effects of cobalt protoporphyrin (CoPP), a HO‐1 inducer, against oxidative damage in c‐kit^+^ CSCs; although, its off‐target effects and transient upregulation of HO‐1 made this strategy clinically non‐feasible (Li et al., 2021). Probucol is a rarely used cholesterol‐lowering drug with HO‐1 inhibitory properties that rescues EPCs against environmental contaminants including cigarette smoke and ambient particulate matter (PM_2.5_), a mixture of crystals, metals, and bio‐aerosols which threat cardiovascular health. It also improves neovascularization in ischemic state through the inhibition of ROS production and apoptosis (Chen et al., 2018). It was also previously discovered, in a rabbit model of balloon‐damaged arteries, that Probucol can stimulate the regeneration of functional ECs and inhibit intimal thickening by activating NO‐cGMP pathway (Lau et al., 2003). Furthermore, the natural *Ginkgo biloba* extract activates EPCs, suppresses the migration of smooth muscle‐like progenitor cells (SMPCs), and enhances vascular protection in atherosclerosis (Wu et al., 2019).

### 7.3 H_2_S‐releasing compounds

Hydrogen sulfide (H_2_S) is a gaseous signaling molecule that exerts a marked protection from oxidative and inflammatory damages and potentiates adaptive responses in the cardiovascular system. H_2_S‐releasing compounds possess a great potential for proliferation of CPC and vascular repair (Zhang et al., 2018). In a mouse model of carotid artery injury, transplantation of NaHS‐preconditioned human EPCs enhanced the capacity of re‐endothelialization, in which H_2_S upregulates the AMPK/eNOS signaling (Ke et al., 2017; Khanna et al., 2020). A series of H_2_S‐releasing compounds including novel synthetic GaOS+PFM, GSGa, and GYY4137 could increase the human CSC proliferation and function in the presence of oxidative stress (Cacciotti et al., 2018; Di Giovanni et al., 2020).

### 7.4 Statins

Recent clinical trials suggest that statins possess a lipid‐lowering‐independent advantage in terms of neovascularization and cardiac regeneration (Cianflone, Cappetta, et al., 2020; Cianflone, Torella, et al., 2020). In an in vivo model of myocardial infarction, a novel formulation involving simvastatin nanoparticles loaded into human adipose‐derived stem cells (hAdSCs) demonstrated pleiotropic effects in BALB/c nude mice subjected to the left anterior descending (LAD) coronary artery ligation. These effects included the improvement of cardiac function and the induction of endogenous cardiac regeneration within the infarcted myocardium. Simvastatin nanoparticles notably increased stem cell proliferation and migration, and most notably, enhanced the differentiation of hAdSCs into immature *Nkx2.5* ^+^/*GATA4* ^+^ cardiomyocytes, as well as endothelial and smooth muscle cells. This led to a reduction in the fibrotic area and an improvement in the echocardiographic profile (Yokoyama et al., 2019). Furthermore, among other statins, rosuvastatin appears to augment the population of endogenous CSCs in rats with myocardial infarction. Similarly, in vitro treatment of CSCs with rosuvastatin and other statins such as simvastatin and pravastatin enhances cell survival and provides protection against H_2_O_2_‐induced apoptosis. This protective mechanism is mechanistically dependent on Akt signaling (Cianflone, Cappetta, et al., 2020; Cianflone, Torella, et al., 2020). It was earlier discovered that another extensively studied statin, atorvastatin, has the capacity to therapeutically increase nitric oxide bioavailability. This is pivotal for enhancing EPC mobilization and facilitating myocardial neovascularization in eNOS^−/−^ mice with extensive anterior myocardial infarction. This interventional approach has the potential to alleviate left ventricular dysfunction and reduce interstitial fibrosis, ultimately contributing to improved survival (Landmesser et al., 2004). Despite the beneficial effects of atorvastatin on cardiac and endothelial progenitor cells, this drug exhibits contradictory effects on hiPSCs. For example, atorvastatin has been observed to diminish the survival of these cells by suppressing the HIF‐1α‐PPAR axis, a crucial factor in maintaining stem cell pluripotency (Nakashima et al., 2018).

### 7.5 Other redox‐regulating compounds with regenerative potential

There is a set of additional redox‐regulating compounds with promises in cardiovascular regeneration. As shown in Table 1, natural active ingredients, including the Chinese herb *Psoralea corylifolia Linn*‐derived Bavachalcone (Ling et al., 2017) and a natural carotenoid lycopene (Zeng et al., 2017), may maintain redox homeostasis and protect EPC against oxidative and advanced AGE‐induced apoptosis, and thereby enhance neovascularization in ischemic and diabetic states. *Rhizoma Polygonati* is another Chinese herb that regulates ROS production in BM‐EPCs and rescues cellular senescence. Its extract reduced SA‐β‐galactosidase positive cells and restores cell proliferation and angiogenic capacity in cultured EPCs and improved their function in aged rats (Qin, 2019). Melatonin also exhibit anti‐aging responses in the cardiovascular system and cardiomyogenic effects markedly through the regulation of emerging epigenetic modulators sirtuins, which are called the “fountain of youth,” LncRNAs or miRNAs (Ren & Zhang, 2018), fostering antioxidant response in cardiac stem cells (Hardeland, 2019). For example, melatonin modulates LncRNA H19/miR‐675/USP10 pathway or miRNA‐98 necessary for cell apoptosis and thereby delays oxidative‐induced senescence (Hardeland, 2019; Ma et al., 2018). Trace element selenium is another natural compound that is capable of promoting vascular progenitor cell differentiation essential for a proper vascular repair. This effect is attributed to its antioxidant response by NOX4 enzyme inhibition and Trx activation. Moreover, selenoproteins may predominantly maintain redox homeostasis in adipose progenitor cells, by which control their proliferation and differentiation. These suggest that selenoproteins are crucial for adipocyte function and their downregulation causes obesity and metabolic disease (Tinkov et al., 2020).

Polyunsaturated fatty acids (PUFAs) represent natural compounds that exert diverse effects on angiogenic and cardiomyogenic processes. Recent investigations have indicated that ω‐3 PUFAs and a diet enriched with fish oil can promote angiogenesis and enhance EPC function, thereby facilitating postnatal neovascularization. However, it is worth noting that ω‐3 PUFAs were initially reported to have anti‐angiogenic effects (Tsuji et al., 2003; Tsuzuki et al., 2007). Notably, a limited number of clinical studies have demonstrated the efficacy of PUFAs in vascular regeneration. The Phase‐IV IPE‐PREVENTION trial (NCT04562467) is currently ongoing to investigate whether and how icosapent ethyl, a pure byproduct of eicosapentaenoic acid (EPA), can increase the formation and functionality of human EPC colonies (CD34^+^/CD133^+^/VEGFR2^+^ cells), thereby reducing EPC depletion and dysfunction. The primary endpoint, which is an increase in circulating EPCs and a shift in the M1/M2 macrophage balance toward a regenerative blood vessel phenotype, is yet to be determined (National Library of Medicine, 2020). Nonetheless, prior investigations have shown that PUFAs primarily suppress oxidative stress. Consequently, preconditioning embryonic stem cell‐derived cardiac lineages with a combination of EPA, docosahexaenoic acid (DHA), and ascorbic acid has enhanced their viability and expression of HO‐1 in response to oxidative stress induced by H2O2. This preconditioning also reduced fibrosis in the ischemic myocardium after transplantation in a rat model of myocardial infarction (Shabani et al., 2019). In contrast, EPA (ω3) and linoleic acid (ω6) have been found to increase NOX‐mediated ROS production and activate endothelial nitric oxide synthase (eNOS), thereby promoting vascular differentiation in mouse embryonic stem cells (Taha et al., 2020).

### 7.6 Senolytics

A novel cell‐free cardiovascular regenerative strategy involves specific small molecules referred to as senolytics, targeting senescent stem/progenitor cells that impede tissue repair during aging. Recent studies confirm senolytics' potential to rejuvenate the regenerative/reparative capacity of aging cardiovascular tissues. Given that senescent cells produce excessive ROS and experience elevated levels of oxidative mitochondrial damage, ROS‐induced SCAP mechanisms can pose challenges by hindering senescent cell elimination. By increasing ROS production, senescent cells enhance their resistance to apoptosis and activate pro‐survival networks (Lewis‐McDougall et al., 2019). Therefore, it is imperative to target oxidative responses to fully unlock the regenerative potential of senolytic approaches in cardiovascular disease treatment (Owens et al., 2021).

Since 2015, when Zhu and colleagues initially reported the senolytic effects of the FDA‐approved tyrosine kinase inhibitor dasatinib for eliminating senescent adipose progenitor cells, extensive translational, and clinical work on senolytic approaches has been undertaken (Zhu et al., 2015). Another senolytic compound tested for its efficacy in eliminating senescent human endothelial cells is Quercetin. In an in vitro model of oxidative stress, quercetin could reduce the population of senescent adipose progenitor cells by suppressing ROS and inflammatory cytokines, while also upregulating SIRT1. The senotherapeutic strategy led to an enhancement of remaining health‐ and lifespan in old mice (Xu et al., 2018). The combination of dasatinib and quercetin (D + Q) has shown synergistic effects in eliminating senescent cardiac progenitor cells in aged mice, thereby restoring cardiac regeneration and function (Zhu et al., 2015). Not only in aging models, the senolytics have demonstrated the ability to clear senescent CSCs and restore their myogenic differentiation capacity in both human and mouse models of Type 2 diabetes, independent of age, efficiently promoting myocardial regeneration/repair. D + Q efficiently clears senescent c‐kit^+^/CD45^−^/CD31^−^ CPCs in diabetic mice by suppressing oxidative stress and SASP, restoring telomere length and proliferative capacity, and enforcing the apoptotic BCL‐2‐mediated pathway (Marino et al., 2022). Other investigations have elucidated that fisetin and navitoclax, BCL‐2 family inhibitors, either alone or in combination using a “hit‐and‐run” intermittent approach, confer senolytic effects in CPCs, leading to improved heart regeneration in both 24–32 month wild‐type mice and INK‐ATTAC transgenic mice (Lewis‐McDougall et al., 2019).

Despite the significant progress in senolytic approaches for effective regenerative therapy of cardiovascular disease in the elderly, off‐target effects on different cells and tissues limit their clinical utility in aging populations. Time‐specific therapies utilizing the next generation of targeted senolytics within a novel drug delivery system are expected to overcome these current challenges, improving safety and specificity for eliminating senescent stem cells.

### 7.7 Biomaterials modulating redox homeostasis for cardiovascular regeneration

Knowing the fact that redox homeostasis is crucial for cardiovascular regeneration and repair, scientists suggest biomaterials as a promising approach to incorporate endogenous antioxidant enzymes or oxygen‐delivery component to respond to local ROS levels. The biomaterials enable to maintain the redox homeostasis with a careful modulation of the release profile (Zhou et al., 2021). Over the last decade, the earlier approaches utilized biomaterials such as melanin (Zhou et al., 2021), graphene oxide (Choe et al., 2019; Han, Bedarida, et al., 2018; Han, Kim, et al., 2018), fullerenol (Hao et al., 2017), and polyurethane (Yao et al., 2020), by which restore redox balance for myocardial repair. Further, scientists supplemented biomaterials with small‐molecule Nrf2 inducers (such as sulforaphane, auranofin, or t‐BHQ) or HIF inducers to improve the survival and functionality of engineered tissues. Furthermore, the recent advancement in injectable alginate hydrogels, which are three‐dimensional materials capable of absorbing water, as well as solid patches combined with small antioxidant molecules like glutathione and vitamin C, in conjunction with nanoparticle‐based stem cell or gene delivery systems, has significantly enhanced neovascularization and the effectiveness of cardiac regenerative approaches (Sthijns et al., 2018). The encouraging findings suggest that redox‐regulating biomaterials enable to address many of the challenges in cardiovascular tissue engineering and regenerative medicine.

## 8 CONCLUDING REMARKS AND FUTURE PERSPECTIVES

The present review provides a comprehensive overview of the intricate role of the redox system in cardiovascular regeneration. It delves into the potential of targeting the redox machinery to optimize stem cell‐based therapies and cardiovascular reprogramming. Currently, certain cardiovascular regenerative approaches have centered on compounds and small molecules that modulate antioxidant defense, particularly Nrf2 and its downstream antioxidant enzymes. However, we also discuss the time‐ and concentration‐specific role of ROS, particularly H_2_O_2_, in optimal somatic cell reprogramming. The effects of ROS in stem/progenitor cells are partially characterized, and their influence on iPSC reprogramming and cardiovascular regeneration remains controversial and enigmatic. In addition to the redox‐regulating compounds discussed in this review, the combination of genome‐wide association study (GWAS) and CRISPR/Cas9 gene‐editing technology, as well as the development of novel small‐molecule senolytics, hold tremendous potential for efficiently and specifically targeting the redox system in stem cells. This could lead to the repair or replacement of cardiovascular tissue function lost due to aging or disease. These advancements offer hope for elderly individuals suffering from cardiovascular diseases, which claim millions of lives each year.

## AUTHOR CONTRIBUTIONS

S.S.S.S. designed, drafted, and wrote the review. S.S.S.S., J.H.B., M.L., and P.L. contributed to the manuscript and figures. S.S.S.S. and J.H.B. obtained the funding.

## CONFLICT OF INTEREST STATEMENT

The authors declare no competing interests.
